# Inactivation of *N-Acetylglucosaminyltransferase I* and *α1,3-Fucosyltransferase* Genes in *Nicotiana tabacum* BY-2 Cells Results in Glycoproteins With Highly Homogeneous, High-Mannose *N*-Glycans

**DOI:** 10.3389/fpls.2021.634023

**Published:** 2021-01-27

**Authors:** Xavier Herman, Johann Far, Adeline Courtoy, Laurent Bouhon, Loïc Quinton, Edwin De Pauw, François Chaumont, Catherine Navarre

**Affiliations:** ^1^Louvain Institute of Biomolecular Science and Technology, UCLouvain, Louvain-la-Neuve, Belgium; ^2^Mass Spectrometry Laboratory-MolSys, GIGA Proteomics Facility, University of Liège, Liège, Belgium

**Keywords:** glyco-engineering, plant suspension cells, oligomannose *N*-glycans, CRISPR/Cas9, molecular farming, *N*-acetylglucosaminyltransferase I, α1, 3-fucosyltransferase

## Abstract

*Nicotiana tabacum* Bright Yellow-2 (BY-2) suspension cells are among the most commonly used plant cell lines for producing biopharmaceutical glycoproteins. Recombinant glycoproteins are usually produced with a mix of high-mannose and complex *N*-glycans. However, *N*-glycan heterogeneity is a concern for the production of therapeutic or vaccine glycoproteins because it can alter protein activity and might lead to batch-to-batch variability. In this report, a BY-2 cell line producing glycoproteins devoid of complex *N*-glycans was obtained using CRISPR/Cas9 edition of two *N-acetylglucosaminyltransferase I (GnTI)* genes, whose activity is a prerequisite for the formation of all complex *N*-glycans. The suppression of complex *N*-glycans in the *GnTI*-knocked out (KO) cell lines was assessed by Western blotting. Lack of β1,2-xylose residues confirmed the abolition of GnTI activity. Unexpectedly, α1,3-fucose residues were still detected albeit dramatically reduced as compared with wild-type cells. To suppress the remaining α1,3-fucose residues, a second genome editing targeted both *GnTI* and α*1,3-fucosyltransferase* (*FucT*) genes. No β1,2-xylose nor α1,3-fucose residues were detected on the glycoproteins produced by the *GnTI/FucT*-KO cell lines. Absence of complex *N*-glycans on secreted glycoproteins of *GnTI*-KO and *GnTI/FucT*-KO cell lines was confirmed by mass spectrometry. Both cell lines produced high-mannose *N*-glycans, mainly Man5 (80 and 86%, respectively) and Man4 (16 and 11%, respectively). The high degree of *N*-glycan homogeneity and the high-mannose *N*-glycosylation profile of these BY-2 cell lines is an asset for their use as expression platforms.

## Introduction

Developing a recombinant glycoprotein production platform in plants with simplified and homogenized N-glycan repertoire represents a key step to design new, highly efficient vaccines and therapeutic glycoproteins. Indeed, production of recombinant glycoproteins usually results in a mix of glycoforms that can display different levels of activity or immunogenicity (Sola and Griebenow, 2010; Lavine et al., 2012; Hariharan and Kane, 2020). Identifying and expressing the most efficient glycoforms could also improve the quality of the product as well as simplify the control of batch-to-bach reproducibility. Impairing the synthesis of complex *N*-glycans, responsible for most of the *N*-glycan diversity, in order to produce only high-mannose *N*-glycans (Man4–Man9) is a powerful strategy to homogenize the *N*-glycan repertoire. *N*-acetylglucosaminyltransferase I (GnTI) is a key Golgi-resident enzyme essential for the processing of high-mannose to hybrid and complex *N*-glycans (Supplementary Figure 1). GnTI transfers an *N*-acetylglucosamine (GlcNAc) residue from UDP-GlcNAc to the acceptor substrate Man5 to produce GnMan5, which is a prerequisite for the subsequent action of all other processing enzymes (Strasser, 2016). The GnTI single-pass transmembrane domain is responsible for the steady-state distribution in the cis/medial-Golgi (Essl et al., 1999; Schoberer et al., 2014, 2019). Since GnTI is required to generate all complex *N*-glycans, inactivation of GnTI in plants has been carried out. It allows *N*-glycan repertoire simplification of produced recombinant glycoproteins and also the suppression of all non-human residues, i.e., β1,2-xylose and α1,3-fucose found on complex *N*-glycans. Another approach to produce recombinant glycoproteins with unprocessed high-mannose *N*-glycans is to prevent trafficking through the Golgi by the addition of a *C*-terminal H/KDEL sequence (Fujiyama et al., 2009).

The first *GnTI* mutants in plants were isolated in *Arabidopsis thaliana* from a pool of random mutagenized seeds (von Schaewen et al., 1993). The *complex glycan-less1 allele 1 (cgl1-1)* mutant lacks GnTI activity and cDNA sequencing identified the point mutation Asp144Asn, which introduced an extra *N*-glycosylation site impairing the GnTI folding (Strasser et al., 2005; Frank et al., 2008). Early reports of the *cgl1-1* mutant did not detect any complex *N*-glycans on glycoproteins of plant extracts, with the predominant type of glycans being Man5 (75%) (Strasser et al., 2005). However, the *cgl1-1* mutant enzyme recovers its activity when the wrong *N*-glycosylation site is skipped (e.g., under stress, in certain developmental stages, or in the *stt3a* background, for the latter see Frank et al., 2008). *A. thaliana cgl1-1* plants growth and morphology were not affected under normal conditions but were more impacted by salt stress than wild-type (WT) plants (Kang et al., 2008). A decrease in photosynthetic capacity of the *cgl1-1* mutant was also reported (Jiao et al., 2020). Many enzymes used as therapeutics for lysosomal storage diseases require phosphorylated high-mannose *N*-glycans for their targeting to the lysosomes (Grubb et al., 2010). Hence, the human lysosomal enzymes glucocerebrosidase and α-L-iduronidase were produced in *cgl1-1* seeds and showed a large proportion of high-mannose-type *N*-glycans (85–94%), predominantly Man5. Nonetheless, significant amounts of MMXF (9–3.8%), Man5F (3.1–0.4%), GnMXF (2.7–0%) or MMX(0–1.3%) on specific *N*-sites were also produced, suggesting the presence of a residual GnTI activity in these seeds (He et al., 2011, 2013; Pierce et al., 2017). The *cgl1-2* mutant (translational frameshift) also lacked GnTI activity (von Schaewen et al., 1993; Frank et al., 2008). Analysis of the activation associated secretory protein 1 produced in *cgl1-2* seeds showed only high-mannose glycans (Piron et al., 2015), suggesting a total loss of GnTI activity.

The rice *gnt1* mutant line (SAI3G12) contains a T-DNA insertion in the sixth exon/intron junction of *GnTI* leading to the absence of native *GnTI* transcripts (Fanata et al., 2013). As a result, only high-mannose *N*-glycans were identified. In contrast with the *A. thaliana cgl1* mutants, rice *gnt1* plants displayed a severe growth phenotype, resulting in early lethality under normal culture conditions. These plants showed a reduction in the cell wall thickness and cellulose content and were insensitive to cytokinin signaling. A rice suspension line was derived from a *gnt1* plant and used to produce the recombinant human acid α- or β-glucosidase. Only mannosylated *N*-glycans were detected on the two recombinant proteins secreted in the culture medium, with a high proportion of Man5 (58% for α-glucosidase and 80% for β-glucosidase) (Jung et al., 2017, 2019).

Several mutants in key enzymes of the *N*-glycosylation pathway were selected from the *Lotus japonicus* retrotransposon 1 insertion population (Pedersen et al., 2017). The *Ljgnt I* mutated plants showed a severely altered growth phenotype, as they predominantly died before the flowering stage. Minimizing the biotic and abiotic stress increased the number of *Ljgnt I* mutants entering the reproduction stage. These results confirmed the differences in tolerance to *GnTI* inactivation between plant species and highlighted the implication of complex *N*-glycans in stress resistance.

*GnTI* knock down in *Nicotiana benthamiana* plants were generated in two different studies by RNAi-mediated silencing. In the first one, GnTI activity was below the detection limit (3%) in one line but sufficient for the synthesis of complex *N*-glycans in amounts comparable to WT plants as no impact on the *N*-glycan repertoire was observed. Complex *N*-glycans still accounted for 80% of total *N*-glycans (Strasser et al., 2004a). It was hypothesized that low *GnTI* expression and activity were sufficient to maintain complex *N*-glycans synthesis. On the other hand, transgenic plants showing a strong reduction in complex *N*-glycans abundance (with only 9% of all *N*-glycan structures as compared to 89% in WT plants) were obtained in the second study (Limkul et al., 2016). No altered growth phenotype was observed in those plants when compared to WT plants. Glycoproteomics analysis of the purified glucocerebrosidase expressed in this mutant background showed a large proportion of high-mannose-type *N*-glycans (73–85%), predominantly Man5, on the four *N*-sites, but also the presence of significant amounts of paucimannose *N*-glycans with β1,2-xylose and α1,3-fucose residues, suggesting a residual GnTI activity (Limkul et al., 2015, 2016).

Reduction of *NtGnTI* activity in *Nicotiana tabacum* was obtained by gene silencing using the antisense technology and evaluated by immunoblot analysis using a complex glycan antiserum on leaf extracts. Transformants showed substantial reduction in complex glycan detection compared with untransformed controls but the reduction of intensity varied according to the leaf age and developmental stage (Wenderoth and von Schaewen, 2000).

Preventing *GnTI* expression in plants thus results in altered phenotype at least under stress conditions. Suspension cells might offer an interesting alternative for the expression of recombinant glycoproteins. *N. tabacum* Bright Yellow-2 (BY-2) suspension cells are characterized by a short culture cycle, easy scaling up in bioreactors, low risk of contamination by human pathogens and the possibility to purify secreted recombinant proteins directly from the culture medium (Tekoah et al., 2015; Santos et al., 2016). BY-2 cells have already been used to express several pharmaceutical glycoproteins such as antibodies (De Muynck et al., 2009; Holland et al., 2010; Magy et al., 2014), a viral glycoprotein (Smargiasso et al., 2019) or human enzymes (Hanania et al., 2017; Ilan et al., 2017). In this study, we report on the identification and characterization of two *GnTI* genes in the *N. tabacum* BY-2 genome. Both were inactivated through a multiplex CRISPR/Cas9 gene edition, which resulted in the non-detection of complex *N*-glycans to the benefit of high-mannose *N*-glycans, mainly the glycoform Man5. However, traces of α1,3-fucose residues were detected on glycoproteins by Western blotting. To eliminate thoroughly this non-human residue, *GnTI* and *fucosyltransferase* (*FucT*) genes were inactivated simultaneously. This combined inactivation resulted in a BY-2 cell line with high-mannose *N*-glycans and no detectable α1,3-fucose. Generation of this BY-2 cell line capable of producing glycoproteins with a high-mannose profile is a powerful tool to improve our understanding of the impact of those *N*-glycans on the properties of vaccinal and biotherapeutic glycoproteins.

## Materials and Methods

### *N. tabacum* BY-2 Cell and Nicotiana Plants Culture

*N. tabacum* cv. BY-2 suspension cells (Nagata et al., 1992) were grown in the dark at 25°C with agitation on a rotary shaker (90 rpm) in liquid MS medium [4.4 g/L Murashige and Skoog salts (MP BIOMEDICALS, Solon, OH, United States), 30 g/L sucrose, 0.2 g/L KH_2_PO_4_, 2.5 mg/L thiamine, 50 mg/L myo-inositol, and 0.2 mg/L 2,4-dichlorophenoxyacetic acid, pH 5.8 (KOH)]. Cultures were grown in 50 mL of medium in a 250 mL Erlenmeyer flask and an 8% inoculum was transferred each week into fresh medium. Transformed cells were grown on MS agar plates supplemented with 100 μg/mL kanamycin. Samples used for genomic analyses and Western blot screening were harvested from liquid cultures obtained after at least two passages on solid selective medium (1–2 months) and at least two passages in liquid selective medium. Lines used for MS analyses were checked by Western blotting and the same profile was invariably observed.

*Nicotiana tomentosiformis*, *N. sylvestris*, and *N. tabacum* seeds were purchased from Bergerac Seed and Breeding. They were germinated and grown in soil under controlled conditions (25°C, 16 h photoperiod).

### Extraction of Genomic DNA

Genomic DNA was extracted from 7-day-old BY-2 cells filtered on four layers of Miracloth (Calbiochem) or from plant leaf material using the Wizard Genomic DNA Purification Kit (Promega).

### Characterization of *GnTI* Orthologs and Analysis of Genome Modifications

Genomic DNA was amplified by PCR using Q5 High-Fidelity DNA Polymerase (New England Biolabs) and the primers listed in Supplementary Table 1. The PCR products were separated by gel electrophoresis on 1% agarose gel, purified, cloned into the pGEM-T Easy vector (Promega), and sequenced.

### RNA Extraction and RT-PCR

RNA extraction was carried out using the Spectrum Plant Total RNA Kit (Sigma). cDNAs were obtained using M-MLV Reverse Transcriptase (Promega). PCR amplification was performed with Q5 High-Fidelity DNA Polymerase (New England Biolabs) and the primers listed in Supplementary Table 1. PCR products were purified and sequenced.

### *GnTI* and *FucT* Gene Accessions

GenBank mRNA accessions are: *NtGnTI.A* (AJ249882), *NtGnTI.B* (AJ249883), *NsGnTI* (XM_009803697 and XM_009776507), *NtoGnTI* (XM_009610507), *FucTA* (XM_016657530), *FucTB* (XM_016620229), *FucTC* (NM_001324945), and *FucTD* (XM_016585847).

### Cas9 and gRNA Binary Plasmid Construction

The four guide RNAs (gRNAs) targeting *GnTI* were obtained by fusing the CRISPR RNA (crRNA) sequence (Supplementary Table 2) to the *trans*-activating crRNA (tracrRNA) optimized by Dang et al. (2015). These gRNAs target exons 1, 3, 13, and 14 at sites conserved in all identified transcript variants of the two *NtGnTI* orthologs. crRNAs were chosen to minimize the risk of off-targets using the Cas-OFFinder tool (Bae et al., 2014). Moreover, crRNAs with high minimum free energy (−1 to 0 kcal/mol) were selected since crRNA secondary structures can greatly hinder edition efficiency. A transfer RNA (tRNA) was placed before each gRNA to generate a polycistronic tRNA-gRNA (PTG) under the control of the *A. thaliana* U6 promoter (Xie et al., 2015).

**FIGURE 1:**
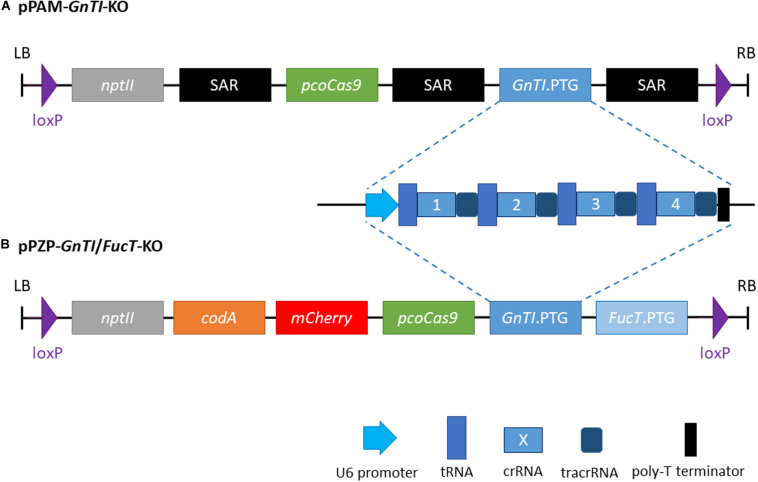
Schematic representation of the two plant transformation vectors. **(A)** pPAM-*GnTI*-KO **(B)** pPZP-*GnTI*/*FucT*-KO. SAR, *N. tabacum* Rb7 scaffold attachment region (genetic insulator); *nptII*, *neomycin phosphotransferase II*; *pcoCas9*, plant codon-optimized *cas9*; PTG, polycistronic tRNA-gRNA; *codA*, *cytosine deaminase A*.

The polycistronic tRNA-gRNA targeting *GnTI* (*GnTI.*PTG) flanked with *Stu*I and *Xho*I restriction sites, was synthesized (Genscript) and cloned into a pUC57 vector. The *FucT/XylT.*PTG of pPAM-FX-KO (Jansing et al., 2019) was replaced by the *GnTI.*PTG using *Stu*I-*Xho*I digestion to generate the pPAM-*GnTI*-KO plasmid, which also contains a plant codon optimized *cas9* with a potato IV2 intron controlled by a hybrid *35SPPDK* promoter (Li et al., 2013) and the selectable marker gene *nptII* (Figure 1A). Cassettes are separated from each other by a *N. tabacum* Rb7 scaffold attachment region (SAR) genetic insulator. Complete sequence between left and right borders is given in Supplementary Figure 2.

To generate *GnTI/FucT*-KO cell lines, a pPZP-RCS2 binary plasmid (Goderis et al., 2002) containing the knockout constructs was prepared (Figure 1B). The pPZP-*GnTI*/*FucT*-KO contains *pcocas9*, *nptII*, *mCherry* reporter gene, the negative selectable marker *cytosine deaminase A* (Stougaard, 1993), the two PTG and loxP sites at both ends of the T-DNA. *GnTI.*PTG obtained above and *FucT.*PTG (Mercx et al., 2017) were cloned into I-CeuI and PI-PspI restriction sites, respectively. Complete sequence between left and right borders is given in Supplementary Figure 3.

### BY-2 Cell Transformation

Biolistic particle delivery was carried out with the plasmid pPAM-*GnTI*-KO. Four milliliters of three-day-old BY-2 cells were vacuum filtered on a Whatman n°4 filter and the cells were transferred on solid MS medium. The next day, plasmid DNA (2 μg) was precipitated on 600 μg of 0.6 μm diameter gold beads (Bio-Rad) and the cells on the filter were bombarded under a 28-inch Hg vacuum (95 kPa), using a 1,100 PSI (7,600 kPa) rupture disk, at a shooting distance of 4 cm with a Biolistic PDS1000/He device (Bio-Rad). The filter was kept on solid MS medium for three days at 25°C under dark conditions. Filters were then transferred onto kanamycin-supplemented MS medium. After four to five weeks, the growing calli were transferred on new solid kanamycin-supplemented MS medium.

*Agrobacterium tumefaciens* LBA4404VirG-mediated transformation of BY-2 cells was carried out with the plasmid pPZP-*GnTI*/*FucT*-KO as described in Navarre et al. (2006).

### SDS-PAGE and Western Blotting Analysis of Proteins

For extracellular protein glycosylation analysis by Western blot, 2 mL of a 7-day-old BY-2 culture in MS medium were filtered on three layers of Miracloth (Calbiochem) and 40 μL of the filtrate were directly analyzed by reducing SDS-PAGE. Filtered BY-2 cells were used to harvest total soluble cellular proteins (TSCPs). Cell packs were transferred into a 2 mL micro tube (Sarstedt) containing 0.5 g of glass beads (0.85–1.23 mm) and 700 μL of homogenization buffer (250 mM sorbitol, 60 mM Tris–HCl, 2 mM Na_2_EDTA, pH 8.0) supplemented with 1 mM phenylmethylsulfonylfluoride (PMSF), and protease inhibitor cocktail (leupeptin, aprotinin, antipain, pepstain, and chymostain, each at 2 μg/mL). Cell grinding was performed for 3 × 40 s at 5,000 rpm (PrecellysTm24 Control Device, Bertin Technologies) with 2 min pauses on ice. The samples were centrifuged first for 5 min at 5,000 rpm at 4°C (Eppendorf Centrifuge 5417C), then for 7 min at 9,400 rpm at 4°C, and finally for 15 min at 54,000 rpm at 4°C (Optima MAX ultracentrifuge, Beckman Coulter). The protein concentration in the supernatant was quantified by Bradford assay and 12 μg TSCP were analyzed by reductive SDS-PAGE (4–20% polyacrylamide). Gels were either stained with Coomassie Brilliant Blue G-250 (SERVA, Heidelberg, Germany) or transferred onto a PVDF membrane (Millipore, Billerica, MA, United States) for Western blotting. The PVDF membrane was incubated with the primary antibodies against β1,2-xylose (monoclonal antibody, Agrisera AS07 267; dilution 1:5,000) or α1,3-fucose (monoclonal antibody, Agrisera AS07 268; dilution 1:5,000) or a mix of both. Secondary horseradish peroxidase (HRP)-conjugated antibodies against rabbit IgG (dilution 1:10,000) were used for extracellular proteins samples. The signals were analyzed using a Kodak Image Station 4000R (Eastman Kodak company, Rochester, NY, United States). HRP-conjugated antibodies were not used for TSCP analysis because of a strong signal attributed to intracellular endogenous peroxidase activity. Rather, secondary alkaline phosphatase-conjugated antibodies against rabbit IgG (dilution 1:10,000) were used.

#### Sample Preparation for MALDI Mass Spectrometry *N*-Glycan Identification

For the mass spectrometry analysis, cultures were grown in liquid D11b medium (Vasilev et al., 2013) without cyclodextrin for 10 days in 50 mL of medium in 250 mL Erlenmeyer flasks. Extracellular proteins were collected by filtration on three layers of Miracloth (Calbiochem), then centrifuged at 8,000 *g* for 30 min, and precipitated by salting-out as previously described in Smargiasso et al. (2019) with the following minor adaptations: solid (NH_4_)_2_SO_4_ was slowly added to the culture medium with stirring at room temperature to 55% saturation and the solution was then kept for 2 h at 4°C and centrifuged at 3,000 *g* for 40 min at 4°C. The pellet was solubilized in 140 μL of PBS buffer and desalted on a PD-10 filtration column (GE Healthcare, Uppsala, Sweden) equilibrated with PBS buffer.

Ribonuclease B from bovine pancreas was used as a positive control sample and was prepared using the same procedure as for the glycoprotein samples from BY-2 cells. The samples were first centrifuged at 8,000 *g* for 5 min at 4°C, the supernatant was filtered on Amicon 3 kDa and the protein content assessed using the RC DC protein essay kit (Bio-Rad). Reduction of 350 μg proteins was performed in 50 mM (NH_4_)_2_CO_3_ and 10 mM of dithiothreitol at 56°C for 40 min at 650 rpm in a thermoshaker. Then alkylation of cysteinyl residues was performed using 20 mM C_2_H_4_INO at RT for 40 min at 650 rpm using a thermoshaker. 2D clean up purification kit (GE Healthcare Life Sciences) was used while the extra washing step (using wash additive) was skipped. Pellets containing proteins were resolubilized in 50 mM (NH_4_)_2_CO_3_ (pH 7.5) before trypsin digestion (protein:trypsin ratio of 50:1) at 37°C for 16 h at 600 rpm. The samples were freeze-dried using SpeedVac centrifugal evaporator and a second trypsin digestion was performed at 37°C in 80% acetonitrile using 100:1 protein:trypsin ratio for 3 h at 600 rpm. Trypsin was inactivated at 90°C for 2 min.

In order to guarantee the removing of all *N*-glycans of interest, a two-step enzymatic release of the *N*-glycans was performed on the trypsin digested samples using first 3 U per 100 μg proteins of PNGase F (Roche, discontinued product) dissolved in 50 mM (NH_4_)_2_CO_3_ (pH 8) at 37°C for 16 h. Samples were freeze-dried and resolubilized in 48.3 mM C_6_H_8_O_7_ and 103.3 mM NaH_2_PO_4_.H_2_O buffer (pH 5). The second deglycosylation step was performed with 0.2 mU of PNGase A (Roche, discontinued product) per 100 μg proteins at 37°C for 16 h at 650 rpm. Samples were stored at −20°C after freeze-drying using a SpeedVac centrifugal evaporator.

The *N*-glycans fraction was separated from the peptide fraction using Waters Sep-Pack SPE C_18_ cartridges after being reconditioned using 5 mL of pure methanol, then 5 mL of 5% acetic acid, 5 mL of pure isopropanol and finally 15 mL of 5% acetic acid. Samples were loaded on the cartridge after solubilizing the dried residue in 200 μL of 5% acetic acid. *N*-glycans were eluted in the 3 mL of 5% acetic acid mobile phase. All the samples were freeze-dried and kept at −20°C. The *N*-glycans containing fraction was desalted using H-cartridges (PROzyme, Agilent) after being reconditioned following the manufacturer recommendations. Desalted *N*-glycans elution was performed as follows: 3 mL of ultrapure water (discarded), 3 mL of 5% acetonitrile and 0.1% trifluoroacetic acid (discarded), and 4 × 0.5 mL of 50% acetonitrile and 0.1% trifluoroacetic acid. These collected fractions were freeze-dried and kept at −20°C.

The labeling of *N*-glycans was then performed using a mixture of 31.5 mg of 2-aminobenzamide (2-AB) and 31.5 mg of NaBH_3_CN in 650 μL of 10 volumes DMSO: 3 volumes glacial acetic acid. Ten microliters of this mixture was added to the dried *N*-glycan fraction and incubated for 2 h at 65°C under 1,200 rpm agitation using a thermoshaker. The 2-AB labeled *N*-glycans were purified using S-cartridges (PROzyme, Agilent) following the manufacturer recommendations and eluted using 3 × 0.5 mL of ultrapure water in Pyrex tubes. The samples were freeze-dried using centrifugal evaporator and kept at −20°C.

##### MALDI-mass spectrometry identification of 2-AB labeled N-glycans

The purified 2-AB labeled *N*-glycans were resolubilized in 10 μL of 50% acetonitrile spiked with 0.1% formic acid and vigorous vortexed in 0.6 mL Eppendorf tube. One microliter of 2-AB labeled *N*-glycan and 1 μL of 2,5-dihydroxybenzoic acid solution (20 mg/mL of 2,5-dihydroxybenzoic acid in 50% acetonitrile and 0.1% formic acid) were mixed and spotted (dried-droplet method) on a MALDI plate Anchorchip384BC fitted on its plate adapter (Bruker) and dried at RT. MALDI mass spectra were obtained using a SolariX XR 9.4T FT-ICR (Bruker) fitted with the dual ESI/MALDI source and Smartbeam LASER. The mass range was set from 150 to 3,500 m/z using 4 megawords acquisition, resulting in an average mass resolving power (FWMH) of about 150,000 at m/z ≈1,100. Two hundred laser shots per scan in small focus mode were selected at the rate of 2,000 Hz. Red P (Sladkova et al., 2009) was used to optimize the ion transmission of m/z going from 800 to 3,000. The MALDI FT-ICR was mass calibrated using Red P and the peptide calibration standard II (Bruker). Mass calibration was checked using the m/z determination of high mannose *N*-glycans released from ribonuclease B. Several replicates of mass spectra were acquired using 12 scan accumulation mode to attempt the detection of minor *N*-glycans. The data were manually processed using DataAnalysis v5.0 software (Bruker). Only peaks with an intensity higher than 1.4 × 10^6^ were considered. The ion mass lists were manually generated based on the GlycoMod tool (Cooper et al., 2001, 2003) using proton and sodium adducts as potentially detected *N*-glycans^1^. Mass accuracy (0.1 Da) was used to refine the list of identified *N*-glycans and to manually annotate the mass spectra.

## Results

### Identification of *N-Acetylglucosaminyltransferase I* Genes in *N. tabacum*

**FIGURE 2:**
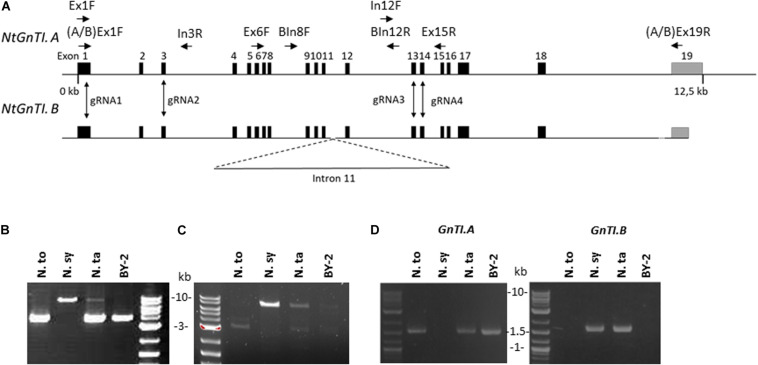
Characterization of Nicotiana *GnTI* genes. **(A)** Schematic representations of *NtGnTI.A* and *NtGnTI.B* genomic sequences. Black rectangles: exons; gray rectangles: 3′UTR; horizontal arrows: primers; vertical arrows: guide RNA target sites; dotted line: interrupted genomic sequence. Note that intron 11 is extended in *NtGnTI.B*. **(B)** PCR amplification of genomic DNA with the non-specific *GnTI* primers [Ex6F and Ex15R]. Bands are expected at 3.8 kb (*GnTI.A*) and 8.6 kb (*GnTI.B*). **(C)** PCR amplification of genomic DNA with *GnTI.B*-specific primers [BIn8F and BIn12R]. *GnTI.B*-specific amplification band is detected at 7.2 kb. **(D)** PCR amplification of cDNAs with *GnTI.A* and *GnTI.B*-specific primers [(A/B)Ex1F and (A/B)Ex19R]. The amplicon size is 1.56 kb. N.to: *N. tomentosiformis*; N.sy: *N. sylvestris*; N.ta: *N. tabacum*.

*N. tabacum* is an allotetraploid species resulting from the cross between *N. tomentosiformis* and *N. sylvestris* (Yukawa et al., 2006). The *NtGnTI* orthologs derived from *N. tomentosiformis* and *N. sylvestris* will be referred to as *NtGnTI.A* and *NtGnTI.B*, respectively, nomenclature previously proposed for *NtSBT1* genes (Navarre et al., 2012), and *NtFucT* or *NtXylT* genes (Mercx et al., 2017). The *NtGnTI.A* and *NtGnTI.B* cDNAs have already been identified from *N. tabacum* cDNA leaf libraries (Strasser et al., 1999; Wenderoth and von Schaewen, 2000). Blast analysis on the four *N. tabacum* genome assemblies (TN90, BX, K326, and Nitab 4.5) as well as the *N. tomentosiformis* and *N. sylvestris* genomes^2^ identified the *GnTI.A* full-length gene in all four *N. tabacum* cultivars (Ntab-TN90_AYMY-SS16267) and in *N. tomentosiformis* (Ntom_KB953441.1). On the other hand, the *GnTI.B* gene was found to be split into three contigs in *N. tabacum* TN90 and BX cultivars (Ntab-TN90_AYMY-SS68155: exons 1-11, Ntab-TN90_AYMY-SS133799: exon 12-18, Ntab-TN90_AYMY-SS242176: exon 19), and in two contigs in *N. sylvestris* (Nsyl_KD955459.1: exons 1-11 and Nsyl_KD976298.1: exons 12-19). Both *NtGnTI* genes were also annotated in NCBI database: *NtGnTI.A* (NCBI gene ID: 107789589) consists of 19 exons spanning on a 12.5 kb genomic sequence (Figure 2A), while *NtGnTI.B* (gene ID: 107800221) is incomplete since the annotated contig covers only the exons 1 to 10.

To compare the *GnTI.A* and *GnTI.B* genomic regions between exons 11 and 12, genomic DNA was extracted from the three Nicotiana plants as well as from BY-2 cells. Next, the *GnTI* region spanning exons 6 to 15 was amplified using non-specific primers [Ex6F and Ex15R] (Figure 2B). The expected 3.8 kb amplicon, corresponding to *GnTI.A*, was detected for *N. tomentosiformis*, *N. tabacum* and BY-2 cells. An 8.6 kb amplicon, corresponding to *GnTI.B*, was detected for *N. sylvestris* and, although less intense, for *N. tabacum* but not for BY-2 cells. Amplification of the region spanning intron 8 to intron 12 was carried out using *GnTI.B* specific primers [BIn8F and BIn12R] (Figure 2C). Sequencing analysis showed that introns 11 of *NtGnTI.B* (Supplementary Figure 4) and *NsGnTI.B* were 5.4 kb long, while introns 11 of *NtGnTI.A* and *NtoGnTI.A* were only 0.4 kb long. *NtGnTI.A* and *NtGnTI.B* intron 11 sequences were aligned and the 5 kb sequence specific to *NtGnTI.B* was blasted on the *N. tabacum* TN90 genome. Two hundred fifty contigs (with an expect score lower than 10^–10^) were identified, showing the presence of numerous sequences highly similar to *NtGnTI.B* intron 11 in the *N. tabacum* genome. *In silico* analysis of *NtGnTI.B* intron 11 sequence revealed the presence of three ORFs of 447, 183, and 507 codons (Supplementary Figure 4), which are associated with the retrovirus-related Pol polyprotein from transposons TNT 1–94, RE1 or RE2 from *Vitis vinifera*. Taken together, these results suggest that the extended intron 11 in *NtGnTI.B* is due to a transposition event.

To assess the expression of both *NtGnTI* orthologs, RNA was extracted from the leaves of the three Nicotiana plants and from BY-2 cells, and analyzed by RT-PCR, using primers specific to each ortholog (Figure 2D). *GnTI.A*-specific primers [AEx1F and AEx19R] amplified the expected 1.56 kb band for *N. tomentosiformis* and *N. tabacum* plants as well as BY-2 cells. On the other hand, *GnTI.B*-specific primers [BEx1F and BEx19R] only gave the expected 1.56 kb band for *N. sylvestris* and *N. tabacum* plants but not for BY-2 cells. This is corroborated by BY-2 cells RNAseq data (Ahmed et al., 2020) in which no cDNA encoding *GnTI.B* was detected. The five *GnTI* cDNAs were sequenced. The coding nucleotide sequences of *NtGnTI.A* and *NtGnTI.B* from *N. tabacum* leaves were identical to the *N. tabacum* cDNAs A4 and A9 already described in the literature (Wenderoth and von Schaewen, 2000). In addition, *NtGnTI.A* sequences obtained from *N. tabacum* leaves and BY-2 cells were identical to the sequence identified in BY-2 cells RNAseq data. Finally, the *NsGnTI* sequence was identical to the database sequences (XM_009803697 and XM_009776507) whereas *NtoGnTI* displayed a single mutation (Thr175Ala), compared to the database sequence (XM_009610507).

### Generation, Screening, and Characterization of BY-2 *GnTI*-KO Cell Lines

**FIGURE 3:**
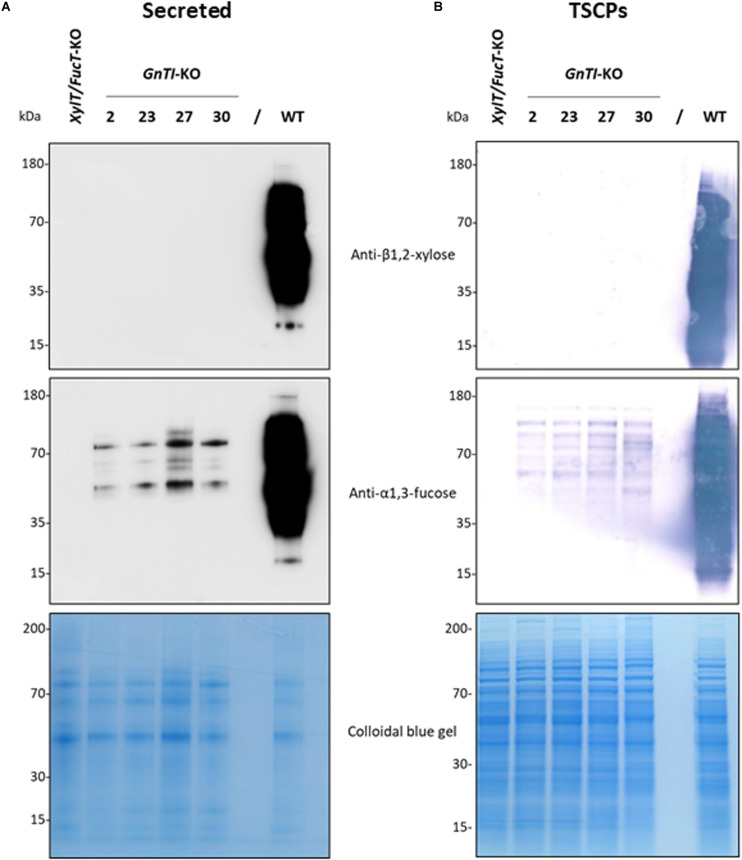
Absence of β1,2-xylose and reduction of α1,3-fucose on glycoproteins from the *GnTI*-KO BY-2 cell lines. Secreted proteins (40 μL culture medium) **(A)** or total soluble cellular proteins (12.5 μg) **(B)** from a WT, *XylT/FucT*-KO, and the indicated *GnTI*-KO cell lines were separated by gel electrophoresis and analyzed by Western blotting using anti-β1,2-xylose or anti-α1,3-fucose antibodies. Horseradish peroxidase-linked or phosphatase alkaline-linked secondary antibodies were used for secreted proteins **(A)** and total soluble cellular proteins **(B)**, respectively. A colloidal blue gel is displayed as a loading control.

Four guide RNAs (gRNAs) were designed to inactivate both *NtGnTI.A* and *NtGnTI.B* by targeting exons 1, 3, 13, and 14 (Supplementary Table 2). A transfer RNA (tRNA) was placed before each gRNA to generate a polycistronic tRNA-gRNA (*GnTI*.PTG). Once the PTG is transcribed, individual gRNAs are expected to be released by the endogenous tRNA processing machinery (Xie et al., 2015). The *GnTI*.PTG construct was cloned into a pPAM plasmid containing a plant codon-optimized Cas9 (Jansing et al., 2019) to generate the pPAM-*GnTI*-KO plasmid (Figure 1A and Supplementary Figure 2). BY-2 cells were transformed with the pPAM-*GnTI*-KO construct using biolistics. After selection on kanamycin-supplemented medium, 36 transgenic lines were screened for *N*-glycosylation profile modification. The extent of complex *N*-glycan suppression was first assessed by analyzing the *N*-glycosylation profile of the proteins secreted in the culture medium by Western blotting combining antibodies recognizing β1,2-xylose and α1,3-fucose epitopes. Complete inactivation of *GnTI* by genome editing was expected to suppress the synthesis of all complex *N*-glycans and thereby incorporation of β1,2-xylose and α1,3-fucose residues. Thirty-one out of 36 cell lines showed a dramatic and similar reduction of complex *N*-glycan-associated signal as compared with the WT cell line (Supplementary Figure 5 and data not shown). However, none of the cell lines displayed a total absence of signals as observed for the *XylT/FucT*-KO cell line used as a control (Mercx et al., 2017). Four *GnTI*-KO lines (*GnTI*-KO#2, 23, 27, 30) were further analyzed using either anti-β1,2-xylose or anti-α1,3-fucose antibodies (Figure 3A). While no signal corresponding to β1,2-xylose could be detected, an α1,3-fucose-specific signal was detected in all four *GnTI*-KO lines, but with a 30 to 100-fold lower intensity compared with the WT cell line. Similar results were obtained when TSCPs were analyzed by Western blotting (Figure 3B).

### Generation, Screening, and Characterization of *GnTI/FucT*-KO Cell Lines

**FIGURE 4:**
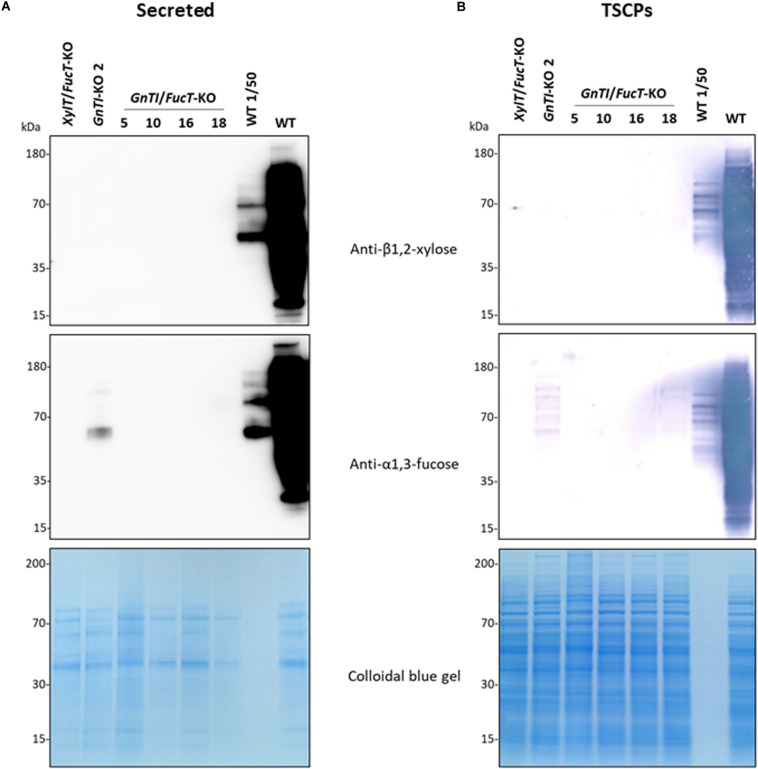
Absence of β1,2-xylose and α1,3-fucose on glycoproteins from *GnTI/FucT*-KO lines. Secreted proteins (40 μL culture medium) **(A)** or total soluble cellular proteins (12.5 μg) **(B)** from WT, *XylT/FucT*-KO, *GnTI*-KO#2, and the indicated *GnTI/FucT*-KO cell lines were separated by gel electrophoresis and analyzed by Western blotting using anti-β1,2-xylose or anti-α1,3-fucose antibodies. Horseradish peroxidase-linked or phosphatase alkaline-linked secondary antibodies were used for secreted proteins **(A)** and total soluble cellular proteins **(B)**, respectively. A 50-fold dilution of WT proteins (equivalent to 0.8 μL culture medium or 0.25 μg TSCPs) were loaded in the penultimate well (WT 1/50). A colloidal blue gel is displayed as a loading control.

To suppress both GnTI and FucT activities, the *GnTI*.PTG genetic construct was combined with the *FucT*.PTG construct previously used to obtain the *XylT/FucT*-KO cell line (Mercx et al., 2017) into a pPZP-RCS2 binary plasmid containing the plant codon-optimized *cas9* (Figure 1B and Supplementary Figure 3). This plasmid is referred to as pPZP-*GnTI*/*FucT*-KO and was used to transform WT BY-2 via *A. tumefaciens* co-cultivation. Preference for Agrobacterium transformation was guided by the need for precise genomic integration if further T-DNA removal was needed, using the Cre-lox system. After selection on kanamycin-supplemented medium, 22 *GnTI/FucT*-KO cell lines were screened using the same immunoblotting strategy as for *GnTI*-KO lines. Fifteen cell lines showed an absence or strong reduction of β1,2-xylose and α1,3-fucose combined signals on secreted glycoproteins (Supplementary Figure 6 and data not shown). Four *GnTI/FucT*-KO cell lines (#5, 10, 16, and 18) for which no signal had been observed were chosen for further analysis. Secreted glycoproteins were analyzed by Western blotting using either anti-β1,2-xylose or anti-α1,3-fucose antibodies (Figure 4A). No β1,2-xylose nor α1,3-fucose residues were detected in any of these lines. Analysis of the TSCPs by Western blotting confirmed the absence of β1,2-xylose and α1,3-fucose residues in glycoproteins of cell lines #5, 10, and 16 (Figure 4B), indicating the complete inactivation of both *GnTI* and *FucT* genes. A faint signal for α1,3-fucose was observed in cell line #18, suggesting that a minor residual FucT activity was still present in that particular cell line.

### Mass Spectrometry Analysis of Total *N*-Glycans on Glycoproteins Secreted by *GnTI-*KO#2 and *GnTI/FucT*-KO#10 Cell Lines

**FIGURE 5:**
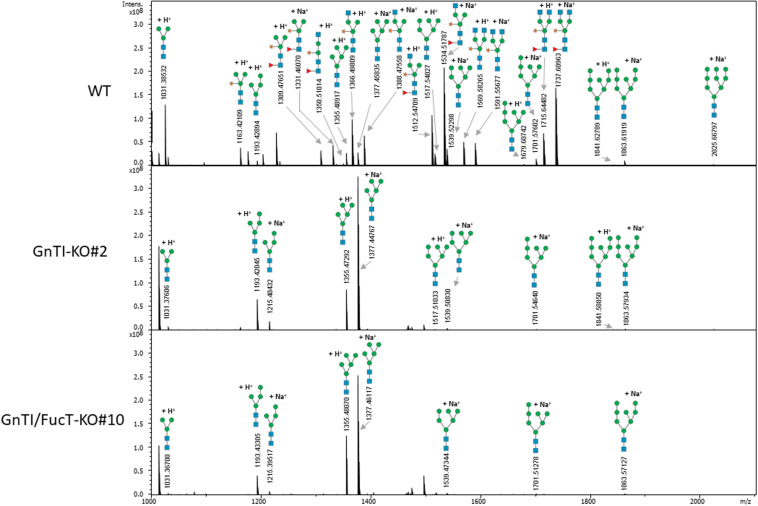
Total 2-aminobenzamide labeled *N*-glycans released from secreted glycoproteins of indicated cell lines. Peaks identified as *N*-glycan signals are labeled. Signals were detected as singly positively charged [M + H]^+^ or [M + Na]^+^ using MALDI FT-ICR mass spectrometry.

Secreted glycoproteins of WT, *GnTI*-KO#2 and *GnTI/FucT*-KO#10 cell lines were analyzed by MALDI-FT ICR mass spectrometry after 2-aminobenzamide reductive labeling to determine their respective *N*-glycoprofile. Spectra are displayed in Figure 5 and relative amounts of the *N*-glycan structures are provided in Table 1. The *N*-glycoprofile of bovine RNAse B was determined as a positive control (Supplementary Figure 7 and Supplementary Table 3). Fourteen *N*-glycan structures were detected on the secreted glycoproteins of WT BY-2 cells. The most abundant structures were GnMXF (27.9%), GnGnXF (20.9%), and GnMX (14.6%). Complex, paucimannosidic and hybrid *N*-glycans accounted for 84.8% of the *N*-glycans on proteins secreted in WT BY-2 cells. High-mannose *N*-glycans made up for only 15.2% of the *N*-glycans. *GnTI* inactivation resulted in a dramatic shift toward high-mannose *N*-glycan structures in *GnTI*-KO#2 and *GnTI/FucT*-KO#10 lines, in which they accounted for 98.4% and 99% of the *N*-glycans, respectively. Only six *N*-glycan structures, corresponding to Man3-8 were identified in the KO lines. The main *N*-glycan structures detected in *GnTI*-KO#2 and *GnTI/FT*-KO#10 were Man5 (79.7 and 85.9%, respectively) and Man4 (16.4 and 11%, respectively).

**TABLE 1:** Relative amounts of total 2-aminobenzamide labeled *N*-glycans (%) decorating the secreted glycoproteins from WT and KO cell lines as determined by MALDI FT-ICR mass spectrometry.

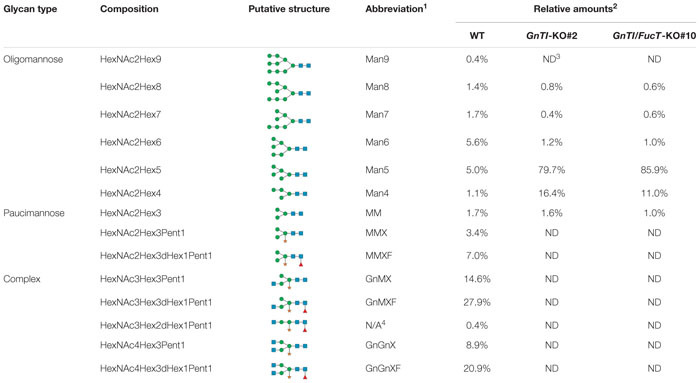

*^1^According to the nomenclature (Proglycan short) from http://www.proglycan.com. Gn, *N*-acetylglucosamine; M/Man, mannose; X, xylose; F, fucose. ^2^Relative amounts were determined by considering only the absolute intensities of the peaks associated to an *N*-glycan structure. ^3^Not detected. ^4^Not assigned.*

### Genomic Analysis of CRISPR/Cas9-Induced Indels in *GnTI*-KO#2 and *GnTI/FucT*-KO#10 Cell Lines

**FIGURE 6:**
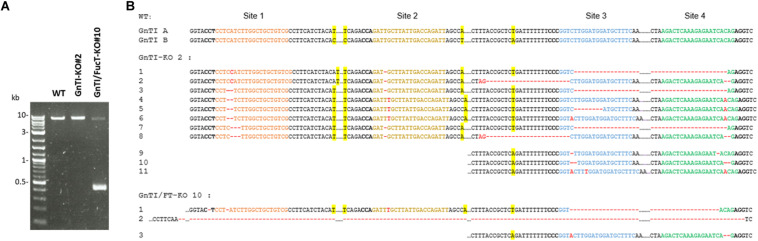
Identification of CRISPR-induced indels in *GnTI* alleles from *GnTI*-KO#2 and *GnTI/FucT*-KO#10 BY-2 cell lines. **(A)** PCR amplification of *GnTI.A* from genomic DNA extracted from the indicated cell lines, using primers [Ex1F and Ex15R]. **(B)** Sequences of the *GnTI* target sites. Colored nucleotides correspond to the gRNA-hybridizing sites. PAM sequences are in bold. CRISPR-Cas9-induced deletions are represented by red dashes, insertions by red nucleotides. Single nucleotide polymorphisms allowing distinguishing *GnTI.A* from *GnTI.B* alleles are highlighted in yellow.

To identify the Cas9-mediated editing of the *GnTI* alleles, genomic DNA was extracted from WT, *GnTI*-KO#2, and *GnTI/FucT*-KO#10 cell lines. The *GnTI* region encompassing the four targeted loci (exon 1 to exon 14) was PCR-amplified using primers hybridizing to conserved regions [Ex1F and Ex15R] (Figure 2A). Gel electrophoresis showed amplicons of the same expected size (7.6 kb) for WT, *GnTI*-KO#2, and *GnTI/FucT*-KO#10 cell lines as well as an extra smaller amplicon (0.44 kb) for *GnTI/FucT*-KO#10 (Figure 6A). Amplicons were cloned and eight different *GnTI.A* alleles in *GnTI*-KO#2 line and two in *GnTI/FucT*-KO#10 were identified by sequencing (Figure 6B). No *GnTI.B* amplicon could be obtained due to the presence of the large 5.4 kb intron 11. To obtain amplicons corresponding to *GnTI.B*, two separate PCR amplifications were carried out to get regions corresponding either to sites 1–2 using primers [Ex1F and In3R] or to sites 3–4 using primers [In12F and Ex15R] (Figure 2A). Amplification of sites 1–2 resulted in obtaining *GnTI.A* sequences exclusively, whereas amplification of sites 3–4 gave both *GnTI.A* and *GnTI.B* sequences. Three *GnTI.B* alleles were identified for *GnTI*-KO#2 and one for *GnTI/FucT*-KO#10 (Figure 6B). Sequence analysis confirmed that these alleles were effectively inactivated. Indeed, three out of the four target sites were mutated in *GnTI.A* allele 4 while all other *GnTI.A* and *GnTI.B* alleles were modified at each observed target site. Most *GnTI*-KO#2 indels consisted of small insertions or deletions but a deletion of 180 nucleotides occurred between sites 3 and 4 in *GnTI.A* alleles 1, 3, and 7. The presence of more than four alleles indicates that some indels were not induced in the initial *A. tumefaciens*-transformed BY-2 cell, but later on in daughter cells. The largest deletion was observed for *GnTI/FucT*-KO#10 cell line. Indeed, the small 0.4 kb amplicon observed in gel electrophoresis (Figure 6A) corresponds to *GnTI.A* allele 2, which underwent a 7.16 kb deletion between sites 1 and 4. All sequences obtained for *GnTI.B* sites 3–4 using primers [In12F and Ex15R] correspond to allele 3, suggesting homozygous mutation. *FucT/GnTI*-KO#10 cell line was likely homogeneous since each *GnTI* ortholog was present in one or two mutated versions.

## Discussion

In this study, we inactivated the two *GnTI* genes in *N. tabacum* BY-2 suspension cells using CRISPR/Cas9 gene edition in order to generate a cell line producing only high-mannose *N*-glycans. GnTI has indeed a key role in the formation of *N*-glycan diversity because it is responsible for the addition of a first GlcNAc, on the α1,3-arm. Addition of this particular residue on a Man5 is considered as a prerequisite before being further modified by Golgi α-mannosidase II, *N*-acetylglucosaminyltransferase II, β1,2-xylosyltransferase, and α1,3-fucosyltransferase to form hybrid, complex and paucimannosidic *N*-glycans (Strasser et al., 2007).

To efficiently knock out *GnTI* in BY-2 cells, we first characterized both *NtGnTI* orthologs. However, *NtGnTI.B* exons 1–11 were detected in *N. tabacum* leaf material but not in BY-2 cells. This suggests that genome modifications impacting the first half of *GnTI.B* occurred in this cell line. It is known that plant suspension cells, including BY-2, might be genetically unstable and subjected to chromosome rearrangements (Kovarik et al., 2012). To guarantee the inactivation of both genes, two pairs of gRNAs targeting the first (exons 1 and 3) and second halves (exons 13 and 14) of *GnTI.A* and *GnTI.B* were designed.

Complete inactivation of *GnTI* was expected to result in a cell line producing only high-mannose *N*-glycans. Western blotting analysis of secreted proteins from the *GnTI*-KO cell lines showed absence of β1,2-xylose residues and an extensive reduction (30-to-100-fold) of α1,3-fucose residues as compared to WT secreted proteins. Absence of *N*-glycans containing β1,2-xylose residues demonstrates the suppression of the entire GnTI activity since GnTI-processed *N*-glycans would be recognized as substrates by Golgi-resident glycosyltransferases like xylosyltransferases (Strasser et al., 2004b; Kaulfurst-Soboll et al., 2011). Genomic analysis of the *GnTI*-KO#2 cell line confirmed the *GnTI* inactivation since all *GnTI* alleles sequenced contained Cas9-induced indels. Complete suppression of α1,3-fucose residues was required because their elimination is desirable to produce glycoproteins lacking non-human epitopes susceptible to trigger allergenic response (van Ree et al., 2000; Altmann, 2007; Gomord et al., 2010). To remove the residual α1,3-fucose, combined inactivation of the two *GnTI* and the four *FucT* genes (twelve alleles) was carried out to generate *GnTI/FucT*-KO cell lines. Secreted and soluble intracellular proteins of three *GnTI/FucT*-KO cell lines (#5, #10, and #16) were totally devoid of any β1,2-xylose and α1,3-fucose residues.

Mass spectrometry analysis of *N*-glycan structures of secreted glycoproteins from WT, *GnTI*-KO#2 and *GnTI-FucT*-KO#10 lines identified the major changes that occurred in the KO lines. While most *N*-glycans in the WT cell line were of complex type, none were detected in either of the KO lines. Man4 and Man5 structures accounted for 96.1 and 96.9% of the *N*-glycans in the *GnTI*-KO#2 and *GnTI*-*FucT*-KO#10 cell lines, respectively. Unlike the Western blotting data, we could not detect any fucosylated structure in the *GnTI*-KO#2 cell line. However, this can be explained by the fact that the α1,3-fucose signal observed by Western blotting in *GnTI*-KO#2 cell line was very weak. It probably belongs to fucosylated *N*-glycans distributed between different masses. Their individual MS signal drops below the detection limit of the method.

Detection of α1,3-fucose on *N*-glycans in *GnTI*-KO cell lines was surprising and can only be explained by a relaxed substrate specificity of FucTs, unlike what is usually reported in the literature. Our hypothesis is that at least one of the four *N. tabacum* FucTs can recognize and process high-mannose *N*-glycans, although with a lower rate than hybrid and complex *N*-glycans. The lack of high-mannose *N*-glycans with α1,3-fucose reported in most plant and plant cell studies is probably due to their very low levels. It could also be correlated with a higher affinity of FucTs for complex *N*-glycans. However, detection of Man5F *N*-glycans on human glucocerebrosidase and α-iduronidase produced in the *A. thaliana* seeds of the conditional *cgl1-1* mutant has been reported several times (He et al., 2011; He et al., 2013; Pierce et al., 2017). The authors linked the presence of Man5F to hexosaminidase activities on the hybrid structure GnMan5F. However, it is highly unlikely that only Man5F would be detected if this structure resulted from hexosaminidase processing. Indeed, neither GnMan5 with or without xylose or α1,3-fucose nor the corresponding hexosaminidase-processed structures Man5XF and Man5X were detected in these studies. We rather suggest that the Man5F *N*-glycan observed in the GnTI-deficient host was not the result of hexosaminidase activity but the product of the α1,3-FucT-catalyzed addition of a core fucose on some Man5 *N*-glycan structures present in very large amount because GnTI was absent. Similar loose substrate specificity was described for the mammalian core α1,6-fucosyltransferase (FUT8). Core α1,6-fucosylation by FUT8 in mammals was first believed to be strictly GnTI-dependent because α1,6-fucosylated *N*-glycans identified by mass spectrometry were for the overwhelming majority hybrid or complex types. Furthermore, *in vitro* studies showed that FUT8 requires the presence of an α1,3-arm GlcNAc (but not an α1,6-arm GlcNAc) to use free *N*-glycans as substrates (Calderon et al., 2016). In contrast to this result, *in vivo* observations showed that, in GnTI-deficient CHO and HEK293S cells, high-mannose *N*-glycans could be efficiently core-fucosylated (Lin et al., 1994; Crispin et al., 2006). In addition, recent *in vitro* Man5 fucosylation assays, using protein- or peptide-linked *N*-glycans instead of free *N*-glycans showed that protein- and peptide-linked Man5 could be fucosylated to some extent, according to the glycopeptide or glycoprotein (Yang et al., 2017). We propose a similar loose substrate specificity of some plant α1,3-FucTs for Man5.

*GnTI*-KO and *GnTI/FucT*-KO BY-2 cell lines produced highly homogeneous glycoproteins secreted in the culture medium with only two main *N*-glycan structures (Man4 and Man5). This *N*-glycoprofile is strongly recommended for some therapeutic glycoproteins (Grubb et al., 2010; He et al., 2011; He et al., 2013; Tekoah et al., 2015; Limkul et al., 2016; Pierce et al., 2017). Furthermore, a high *N*-glycan homogeneity can ease the protein purification as well as the process reproducibility, leading to more consistent therapeutic efficacy (Meuris et al., 2014). As a result, the development of a BY-2 production platform with simplified and homogenized *N*-glycan repertoire, such as the humanized *XylT/FucT*-KO cell lines (Hanania et al., 2017; Mercx et al., 2017) or the two glyco-engineered cell lines generated in this study, is an important step to produce highly efficient therapeutic glycoproteins and simplify vaccine production in a system free of potential mammalian pathogens.

## Data Availability Statement

The original contributions presented in the study are publicly available. This data can be found here: Repository MassIVE: MSV000086640, ftp://massive.ucsd.edu/MSV000086640/.

## Author Contributions

XH and CN: conceptualization. XH and JF: methodology. XH, JF, AC, LB, and CN: investigation. XH, JF, and CN: data curation. XH: writing – original draft preparation. CN, JF, and FC: writing – review and editing. CN, LQ, and FC: supervision. FC and EDP: funding acquisition. All authors have read and agreed to the manuscript.

## Conflict of Interest

The authors declare that the research was conducted in the absence of any commercial or financial relationships that could be construed as a potential conflict of interest.

## Footnotes

1 https://web.expasy.org/glycomod/

2 https://solgenomics.net/organism/Nicotiana_tabacum/genome
