# Supplementary material for: Inactivation of N-Acetylglucosaminyltransferase I and α1,3-Fucosyltransferase Genes in Nicotiana tabacum BY-2 Cells Results in Glycoproteins With Highly Homogeneous, High-Mannose N-Glycans
Source: Front Plant Sci. 2021 Jan 27;12:634023. doi: 10.3389/fpls.2021.634023 (PMC7873608; doi:10.3389/fpls.2021.634023)
Supplement: Supplementary file 1 [file Data_Sheet_1.docx]

Supplementary Material

# Supplementary Figures and Tables

## Supplementary Figures


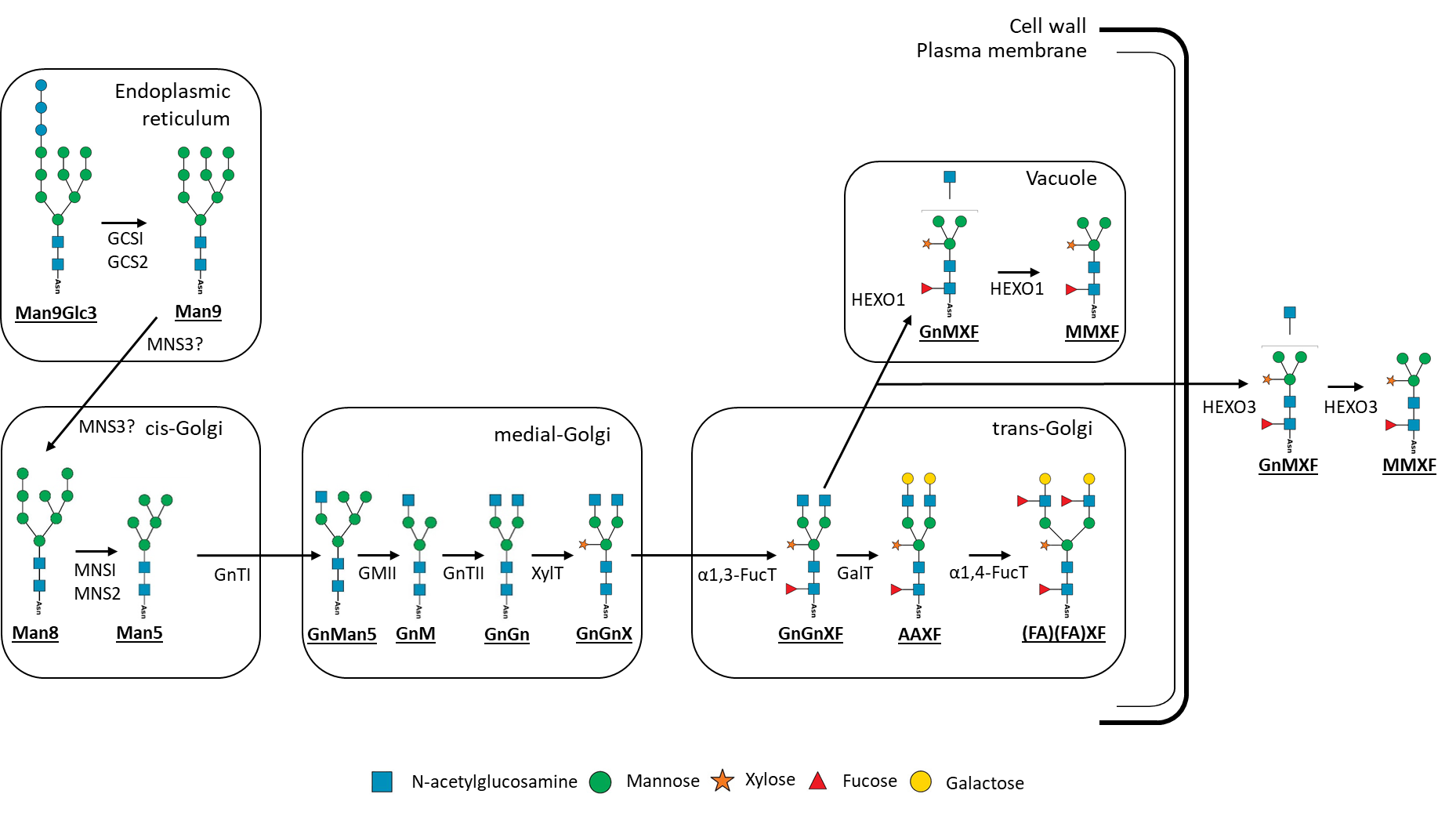


**Supplementary Figure 1.** Schematic plant N-glycosylation pathway. N-glycan abbreviations are used according to the ProGlycan short nomenclature (www.proglycan.com). GCS, α-glucosidase; MNS, class I α-mannosidase; GnT, N-acetylglucosaminyltransferase; GMII, Golgi-α-mannosidase II; XylT, β-1,2-xylosyltransferase; FucT, fucosyltransferase; GalT, galactosyltransferase; HEXO, hexosaminidase. Adapted from Strasser, 2016.

TGGCAGGATATATTGTGGTGTAAACAAATTGACGCTTAGACAACTTAATAACACATTGCGGACGTTTTTAATGATCGAATACTAACGTCTCTACCAGATATCAGCTTGCATGCTTGGCAGGGGGTGGGAGGGAAGGATAACTTCGTATAATGTATGCTATACGAAGTTATGCATGCCGGTCGATCTAGTAACATAGATGACACCGCGCGCGATAATTTATCCTAGTTTGCGCGCTATATTTTGTTTTCTATCGCGTATTAAATGTATAATTGCGGGACTCTAATCATAAAAACCCATCTCATAAATAACGTCATGCATTACATGTTAATTATTACATGCTTAACGTAATTCAACAGAAATTATATGATAATCATCGCAAGACCGGCAACAGGATTCAATCTTAAGAAACTTTATTGCCAAATGTTTGAACGATCTGCTTGACTCTAGCTAGAGTCCGAACCCCAGAGTCCCGCTCAGAAGAACTCGTCAAGAAGGCGATAGAAGGCGATGCGCTGCGAATCGGGAGCGGCGATACCGTAAAGCACGAGGAAGCGGTCAGCCCATTCGCCGCCAAGCTCTTCAGCAATATCACGGGTAGCCAACGCTATGTCCTGATAGCGGTCCGCCACACCCAGCCGGCCACAGTCGATGAATCCAGAAAAGCGGCCATTTTCCACCATGATATTCGGCAAGCAGGCATCGCCGTGGGTCACGACGAGATCCTCGCCGTCGGGCATCCGCGCCTTGAGCCTGGCGAACAGTTCGGCTGGCGCGAGCCCCTGATGCTCTTCGTCCAGATCATCCTGATCGACAAGACCGGCTTCCATCCGAGTACGTGCTCGCTCGATGCGATGTTTCGCTTGGTGGTCGAATGGGCAGGTAGCCGGATCAAGCGTATGCAGCCGCCGCATTGCATCAGCCATGATGGATACTTTCTCGGCAGGAGCAAGGTGAGATGACAGGAGATCCTGCCCCGGCACTTCGCCCAATAGCAGCCAGTTCCTTCCCGCTTCAGTGACAACGTCGAGCACAGCTGCGCAAGGAACGCCCGTTGTGGCCAGCCACGATAGCCGCGCTGCCTCGTCTTGGAGTTCATTCAGGGCACCGGACAGGTCGGTCTTGACAAAAAGAACCGGGCGCCCCTGCGCTGACAGCCGGAACACGGCGGCATCAGAGCAGCCGATTGTTTGTTGTGCCCAGTCATAGCCGAATAGCCTCTCCACCCAAGCGGCCGGAGAACCTGCGTGCAATCCATCTTGTTCAATCATGCCTGCATCGAGTTGAGAGTGAATATGAGACTCTAATTGGATACCGAGGGGAATTTATGGAACGTCAGTGGAGCATTTTTGACAAGAAATATTTGCTAGCTGATAGTGACCTTAGGCGACTTTTGAACGCGCAATAATGGTTTCTGACGTATGTGCTTAGCTCATTAAACTCCAGAAACCCGCGGCTGAGTGGCTCCTTCAACGTGCGGTTCTGTCAGTTCCAAACGTAAAACGGCTTGTCCCGCGTCATCGGCGGGGGTCATAACGTGACTCCCTTAATTCTCCGCTCATGATCGATATCCATTGAAGAGCAAGCTAATTCCGCCCCTAGAAATATTTGCGACTCTTCTGGCATGTAATATTTCGTTAAATATGAAGTGCTCCATTTTTATTAACTTTAAATAATTGGTTGTACGATCACTTTCTTATCAAGCGTTACTAAAATGCGTCAATCTCTTTGTTCTTCCATATTCATATGTCAAAATCTATCAAAATTCTTATATATCTTTTTCGAATTTGAAGTGAAATTTCGATAATTTAAAATTAAATAGAACATATCATTATTTAGGTGTCATATTGATTTTTATACTTAATTACTAAATTTGGTTAACTTTGAAAGTGTACATCAACGAAAAATTAGTCAAACGACTAAAATAAATAAATATCATGTGTTATTAAGAAAATTCTCCTATAAGAATATTTTAATAGATCATATGTTTGTAAAAAAAATTAATTTTTACTAACACATATATTTACTTATCAAAAATTTGACAAAGTAAGATTAAAATAATATTCATCTAACAAAAAAAAACCAGAAAATGCTGAAAACCCGGCAAAACCGAACCAATCCAAACCGATATAGTTGGTTTGGTTTGATTTTGATATAAACCGAACCAACTCGGTCCATTTGCACCCCTAATCATAATAGCTTTAATATTTCAAGATATTATTAAGTTAACGTTGTCAATATCCTGGAAATTTTGCAAAATGAATCAAGCCTATATGGCTGTAATATGAATTTAAAAGCAGCTCGATGTGGTGGTAATATGTAATTTACTTGATTCTAAAAAAATATCCCAAGTATTAATAATTTCTGCTAGGAAGAAGGTTAGCTACGATTTACAGCAAAGCCAGAATACAAAGAACCATAAAGTGATTGAAGCTCGAAATATACGAAGGAACAAATATTTTTAAAAAAATACGCAATGACTTGGAACAAAAGAAAGTGATATATTTTTTGTTCTTAAACAAGCATCCCCTCTAAAGAATGGCAGTTTTCCTTTGCATGTAACTATTATGCTCCCTTCGTTACAAAAATTTTGGACTACTATTGGGCGCGGGGCGCGGGGCGTCGAGAAGCTTACTCCAAGAATATCAAAGATACAGTCTCAGAAGACCAAAGGGCTATTGAGACTTTTCAACAAAGGGTAATATCGGGAAACCTCCTCGGATTCCATTGCCCAGCTATCTGTCACTTCATCAAAAGGACAGTAGAAAAGGAAGGTGGCACCTACAAATGCCATCATTGCGATAAAGGAAAGGCTATCGTTCAAGATGCCTCTGCCGACAGTGGTCCCAAAGATGGACCCCCACCCACAAGGAGCATCGTGGAAAAAGAAGACGTTCCAACCACGTCTTCAAAGCAAGTGGATTGATGTGATATCTCCACTGACGTAAGGGATGACGCACAATCCCACTATCCTTCGCCCCAAGCTTGGGCCCAAGCTTGGGTCGCGCCCCACGGATGGTATAAGAATAAAGGCATTCCGCGTGCAGGATTCACCCGTTCGCCTCTCACCTTTTCGCTGTACTCTCTCGCCACACACACCCCCTCTCCAGCTCCGTTGGAGCTCCGGACAGCAGCAGGCGCGGGGCGGTCACGTAGTAAGCAGCTCTCGGCTCCCTCTCCCCTTGCTCCGTGGATCCATGGATTACAAGGATGATGATGATAAGGATTACAAGGATGATGATGATAAGATGGCTCCAAAGAAGAAGAGAAAGGTTGGAATCCACGGAGTTCCAGCTGCTGATAAGAAGTACTCTATCGGACTTGACATCGGAACCAACTCTGTTGGATGGGCTGTTATCACCGATGAGTACAAGGTTCCATCTAAGAAGTTCAAGGTTCTTGGAAACACCGATAGACACTCTATCAAGAAGAACCTTATCGGTGCTCTTCTTTTCGATTCTGGAGAGACCGCTGAGGCTACCAGATTGAAGAGAACCGCTAGAAGAAGATACACCAGAAGAAAGAACAGAATCTGCTACCTTCAGGAAATCTTCTCTAACGAGATGGCTAAGGTTGATGATTCTTTCTTCCACAGACTTGAGGAGTCTTTCCTTGTTGAGGAGGATAAGAAGCACGAGAGACACCCAATCTTCGGAAACATCGTTGATGAGGTTGCTTACCACGAGAAGTACCCAACCATCTACCACCTTAGAAAGAAGTTGGTTGATTCTACCGATAAGGCTGATCTTAGACTTATCTACCTTGCTCTTGCTCACATGATCAAGTTCAGAGGACACTTCCTTATCGAGGGAGACCTTAACCCAGATAACTCTGATGTTGATAAGTTGTTCATCCAGCTTGTTCAGACCTACAACCAGCTTTTCGAGGAGAACCCAATCAACGCTTCTGGAGTTGATGCTAAGGCTATCCTTTCTGCTAGACTTTCTAAGTCTCGTAGACTTGAGAACCTTATCGCTCAGCTTCCAGGAGAGAAGAAGAACGGACTTTTCGGAAACCTTATCGCTCTTTCTCTTGGACTTACCCCAAACTTCAAGTCTAACTTCGATCTTGCTGAGGATGCTAAGTTGCAGCTTTCTAAGGATACCTACGATGATGATCTTGATAACCTTCTTGCTCAGATCGGAGATCAGTACGCTGATCTTTTCCTTGCTGCTAAGAACCTTTCTGATGCTATCCTTCTTTCTGACATCCTTAGAGTTAACACCGAGATCACCAAGGCTCCACTTTCTGCTTCTATGATCAAGAGATACGATGAGCACCACCAGGATCTTACCCTTTTGAAGGCTCTTGTTAGACAGCAGCTTCCAGAGAAGTACAAGGAAATCTTCTTCGATCAGTCTAAGAACGGATACGCTGGATACATCGATGGAGGAGCTTCTCAGGAGGAGTTCTACAAGTTCATCAAGCCAATCCTTGAGAAGATGGATGGAACCGAGGAGCTTCTTGTTAAGTTGAACAGAGAGGATCTTCTTAGAAAGCAGAGAACCTTCGATAACGGATCTATCCCACACCAGATCCACCTTGGAGAGCTTCACGCTATCCTTCGTAGACAGGAGGATTTCTACCCATTCTTGAAGGATAACAGAGAGAAGATCGAGAAGATCCTTACCTTCAGAATCCCATACTACGTTGGACCACTTGCTAGAGGAAACTCTCGTTTCGCTTGGATGACCAGAAAGTCTGAGGAGACCATCACCCCTTGGAACTTCGAGGAGGTAAGTTTCTGCTTCTACCTTTGATATATATATAATAATTATCATTAATTAGTAGTAATATAATATTTCAAATATTTTTTTCAAAATAAAAGAATGTAGTATATAGCAATTGCTTTTCTGTAGTTTATAAGTGTGTATATTTTAATTTATAACTTTTCTAATATATGACCAAAATTTGTTGATGTGCAGGTTGTTGATAAGGGAGCTTCTGCTCAGTCTTTCATCGAGAGAATGACCAACTTCGATAAGAACCTTCCAAACGAGAAGGTTCTTCCAAAGCACTCTCTTCTTTACGAGTACTTCACCGTTTACAACGAGCTTACCAAGGTTAAGTACGTTACCGAGGGAATGAGAAAGCCAGCTTTCCTTTCTGGAGAGCAGAAGAAGGCTATCGTTGATCTTCTTTTCAAGACCAACAGAAAGGTTACCGTTAAGCAGTTGAAGGAGGATTACTTCAAGAAGATCGAGTGCTTCGATTCTGTTGAAATCTCTGGAGTTGAGGATAGATTCAACGCTTCTCTTGGAACCTACCACGATCTTTTGAAGATCATCAAGGATAAGGATTTCCTTGATAACGAGGAGAACGAGGACATCCTTGAGGACATCGTTCTTACCCTTACCCTTTTCGAGGATAGAGAGATGATCGAGGAGAGACTCAAGACCTACGCTCACCTTTTCGATGATAAGGTTATGAAGCAGTTGAAGAGAAGAAGATACACCGGATGGGGTAGACTTTCTCGTAAGTTGATCAACGGAATCAGAGATAAGCAGTCTGGAAAGACCATCCTTGATTTCTTGAAGTCTGATGGATTCGCTAACAGAAACTTCATGCAGCTTATCCACGATGATTCTCTTACCTTCAAGGAGGACATCCAGAAGGCTCAGGTTTCTGGACAGGGAGATTCTCTTCACGAGCACATCGCTAACCTTGCTGGATCTCCAGCTATCAAGAAGGGAATCCTTCAGACCGTTAAGGTTGTTGATGAGCTTGTTAAGGTTATGGGTAGACACAAGCCAGAGAACATCGTTATCGAGATGGCTAGAGAGAACCAGACCACCCAGAAGGGACAGAAGAACTCTCGTGAGAGAATGAAGAGAATCGAGGAGGGAATCAAGGAGCTTGGATCTCAAATCTTGAAGGAGCACCCAGTTGAGAACACCCAGCTTCAGAACGAGAAGTTGTACCTTTACTACCTTCAGAACGGAAGAGATATGTACGTTGATCAGGAGCTTGACATCAACAGACTTTCTGATTACGATGTTGATCACATCGTTCCACAGTCTTTCTTGAAGGATGATTCTATCGATAACAAGGTTCTTACCCGTTCTGATAAGAACAGAGGAAAGTCTGATAACGTTCCATCTGAGGAGGTTGTTAAGAAGATGAAGAACTACTGGAGACAGCTTCTTAACGCTAAGTTGATCACCCAGAGAAAGTTCGATAACCTTACCAAGGCTGAGAGAGGAGGACTTTCTGAGCTTGATAAGGCTGGATTCATCAAGAGACAGCTTGTTGAGACCAGACAGATCACCAAGCACGTTGCTCAGATCCTTGATTCTCGTATGAACACCAAGTACGATGAGAACGATAAGTTGATCAGAGAGGTTAAGGTTATCACCTTGAAGTCTAAGTTGGTTTCTGATTTCAGAAAGGATTTCCAGTTCTACAAGGTTAGAGAGATCAACAACTACCACCACGCTCACGATGCTTACCTTAACGCTGTTGTTGGAACCGCTCTTATCAAGAAGTACCCAAAGTTGGAGTCTGAGTTCGTTTACGGAGATTACAAGGTTTACGATGTTAGAAAGATGATCGCTAAGTCTGAGCAGGAGATCGGAAAGGCTACCGCTAAGTACTTCTTCTACTCTAACATCATGAACTTCTTCAAGACCGAGATCACCCTTGCTAACGGAGAGATCAGAAAGAGACCACTTATCGAGACCAACGGAGAGACCGGAGAGATCGTTTGGGATAAGGGAAGAGATTTCGCTACCGTTAGAAAGGTTCTTTCTATGCCACAGGTTAACATCGTTAAGAAAACCGAGGTTCAGACCGGAGGATTCTCTAAGGAGTCTATCCTTCCAAAGAGAAACTCTGATAAGTTGATCGCTAGAAAGAAGGATTGGGACCCAAAGAAGTACGGAGGATTCGATTCTCCAACCGTTGCTTACTCTGTTCTTGTTGTTGCTAAGGTTGAGAAGGGAAAGTCTAAGAAGTTGAAGTCTGTTAAGGAGCTTCTTGGAATCACCATCATGGAGCGTTCTTCTTTCGAGAAGAACCCAATCGATTTCCTTGAGGCTAAGGGATACAAGGAGGTTAAGAAGGATCTTATCATCAAGTTGCCAAAGTACTCTCTTTTCGAGCTTGAGAACGGAAGAAAGAGAATGCTTGCTTCTGCTGGAGAGCTTCAGAAGGGAAACGAGCTTGCTCTTCCATCTAAGTACGTTAACTTCCTTTACCTTGCTTCTCACTACGAGAAGTTGAAGGGATCTCCAGAGGATAACGAGCAGAAGCAGCTTTTCGTTGAGCAGCACAAGCACTACCTTGATGAGATCATCGAGCAAATCTCTGAGTTCTCTAAGAGAGTTATCCTTGCTGATGCTAACCTTGATAAGGTTCTTTCTGCTTACAACAAGCACAGAGATAAGCCAATCAGAGAGCAGGCTGAGAACATCATCCACCTTTTCACCCTTACCAACCTTGGTGCTCCAGCTGCTTTCAAGTACTTCGATACCACCATCGATAGAAAAAGATACACCTCTACCAAGGAGGTTCTTGATGCTACCCTTATCCACCAGTCTATCACCGGACTTTACGAGACCAGAATCGATCTTTCTCAGCTTGGAGGAGATAAGAGACCAGCTGCTACCAAGAAGGCTGGACAGGCTAAGAAGAAGAAGTGACTGCAGATCGTTCAAACATTTGGCAATAAAGTTTCTTAAGATTGAATCCTGTTGCCGGTCTTGCGATGATTATCATATATATTTCTGTTGATTACGTTAAGCATGTAATAATTAACATGTAATGCATGACGTTATTTATGAGATGGGTTTTTATGATTAGAGTCCCGCAATTATACATTTAATACGCGATAGAAAACAAAATATAGCGCGCAAACTAGGATAAATTATCGCGCGCGGTGTCATCTATGTTACTAGATCCGATGATAAGCTGTCAAACATGAGAATTCCGCCCCTAGAAATATTTGCGACTCTTCTGGCATGTAATATTTCGTTAAATATGAAGTGCTCCATTTTTATTAACTTTAAATAATTGGTTGTACGATCACTTTCTTATCAAGCGTTACTAAAATGCGTCAATCTCTTTGTTCTTCCATATTCATATGTCAAAATCTATCAAAATTCTTATATATCTTTTTCGAATTTGAAGTGAAATTTCGATAATTTAAAATTAAATAGAACATATCATTATTTAGGTGTCATATTGATTTTTATACTTAATTACTAAATTTGGTTAACTTTGAAAGTGTACATCAACGAAAAATTAGTCAAACGACTAAAATAAATAAATATCATGTGTTATTAAGAAAATTCTCCTATAAGAATATTTTAATAGATCATATGTTTGTAAAAAAAATTAATTTTTACTAACACATATATTTACTTATCAAAAATTTGACAAAGTAAGATTAAAATAATATTCATCTAACAAAAAAAAACCAGAAAATGCTGAAAACCCGGCAAAACCGAACCAATCCAAACCGATATAGTTGGTTTGGTTTGATTTTGATATAAACCGAACCAACTCGGTCCATTTGCACCCCTAATCATAATAGCTTTAATATTTCAAGATATTATTAAGTTAACGTTGTCAATATCCTGGAAATTTTGCAAAATGAATCAAGCCTATATGGCTGTAATATGAATTTAAAAGCAGCTCGATGTGGTGGTAATATGTAATTTACTTGATTCTAAAAAAATATCCCAAGTATTAATAATTTCTGCTAGGAAGAAGGTTAGCTACGATTTACAGCAAAGCCAGAATACAAAGAACCATAAAGTGATTGAAGCTCGAAATATACGAAGGAACAAATATTTTTAAAAAAATACGCAATGACTTGGAACAAAAGAAAGTGATATATTTTTTGTTCTTAAACAAGCATCCCCTCTAAAGAATGGCAGTTTTCCTTTGCATGTAACTATTATGCTCCCTTCGTTACAAAAATTTTGGACTACTATTGGGCGGGTGGAGGGGGATCAGATTGTCGTTTCCCGCCTTCAGTTTCGCGCCCAGAAATCTCAAAATTCCGGCAGAACAATTTTGAATCTCGATCCGTAGAAACGAGACGGTCATTGTTTTAGTTCCACCACGATTATATTTGAAATTTACGTGAGTGTGAGTGAGACTTGCATAAGAAAATAAAATCTTTAGTTGGGAAAAAATTCAATAATATAAATGGGCTTGAGAAGGAAGCGAGGGATAGGCCTTTTTCTAAAATAGGCCCATTTAAGCTATTAACAATCTTCAAAAGTACCACAGCGCTTAGGTAAAGAAAGCAGCTGAGTTTATATATGGTTAGAGACGAAGTAGTGATTGAACAAAGCACCAGTGGTCTAGTGGTAGAATAGTACCCTGCCACGGTACAGACCCGGGTTCGATTCCCGGCTGGTGCACGACAGCAGCCAAGATGAGGGTTTCAGAGCTATGCTGGAAACAGCATAGCAAGTTGAAATAAGGCTAGTCCGTTATCAACTTGAAAAAGTGGCACCGAGTCGGTGCAACAAAGCACCAGTGGTCTAGTGGTAGAATAGTACCCTGCCACGGTACAGACCCGGGTTCGATTCCCGGCTGGTGCAAATCTGGTCAATAAGCAATCGTTTCAGAGCTATGCTGGAAACAGCATAGCAAGTTGAAATAAGGCTAGTCCGTTATCAACTTGAAAAAGTGGCACCGAGTCGGTGCAACAAAGCACCAGTGGTCTAGTGGTAGAATAGTACCCTGCCACGGTACAGACCCGGGTTCGATTCCCGGCTGGTGCAGAAAGCATCCATCCAAGACCGTTTCAGAGCTATGCTGGAAACAGCATAGCAAGTTGAAATAAGGCTAGTCCGTTATCAACTTGAAAAAGTGGCACCGAGTCGGTGCAACAAAGCACCAGTGGTCTAGTGGTAGAATAGTACCCTGCCACGGTACAGACCCGGGTTCGATTCCCGGCTGGTGCAAGACTCAAAGAGAATCACAGGTTTCAGAGCTATGCTGGAAACAGCATAGCAAGTTGAAATAAGGCTAGTCCGTTATCAACTTGAAAAAGTGGCACCGAGTCGGTGCTTTTTTTCTAGACCCAGCTTTCTTGTACAAAGTTGGCATTAGAATATATATATATTCCCAAGCTGGTACCCCGAGTGTCGACCTCGAGGCATGACAATTGGATGGCCGGCCGCCCCTAGAAATATTTGCGACTCTTCTGGCATGTAATATTTCGTTAAATATGAAGTGCTCCATTTTTATTAACTTTAAATAATTGGTTGTACGATCACTTTCTTATCAAGCGTTACTAAAATGCGTCAATCTCTTTGTTCTTCCATATTCATATGTCAAAATCTATCAAAATTCTTATATATCTTTTTCGAATTTGAAGTGAAATTTCGATAATTTAAAATTAAATAGAACATATCATTATTTAGGTGTCATATTGATTTTTATACTTAATTACTAAATTTGGTTAACTTTGAAAGTGTACATCAACGAAAAATTAGTCAAACGACTAAAATAAATAAATATCATGTGTTATTAAGAAAATTCTCCTATAAGAATATTTTAATAGATCATATGTTTGTAAAAAAAATTAATTTTTACTAACACATATATTTACTTATCAAAAATTTGACAAAGTAAGATTAAAATAATATTCATCTAACAAAAAAAAACCAGAAAATGCTGAAAACCCGGCAAAACCGAACCAATCCAAACCGATATAGTTGGTTTGGTTTGATTTTGATATAAACCGAACCAACTCGGTCCATTTGCACCCCTAATCATAATAGCTTTAATATTTCAAGATATTATTAAGTTAACGTTGTCAATATCCTGGAAATTTTGCAAAATGAATCAAGCCTATATGGCTGTAATATGAATTTAAAAGCAGCTCGATGTGGTGGTAATATGTAATTTACTTGATTCTAAAAAAATATCCCAAGTATTAATAATTTCTGCTAGGAAGAAGGTTAGCTACGATTTACAGCAAAGCCAGAATACAAAGAACCATAAAGTGATTGAAGCTCGAAATATACGAAGGAACAAATATTTTTAAAAAAATACGCAATGACTTGGAACAAAAGAAAGTGATATATTTTTTGTTCTTAAACAAGCATCCCCTCTAAAGAATGGCAGTTTTCCTTTGCATGTAACTATTATGCTCCCTTCGTTACAAAAATTTTGGACTACTATTGGGCGGGTGGAGGGGGATCAGATTGTCGTTTCCCGCCTTCAGTTTAAACATAACTTCGTATAATGTATGCTATACGAAGTTATTTGGCAGGGGGTGGGAGGGAAGGTTTAAACTATCAGTGTTTGACAGGATATATTGGCGGGTAAAC

**Supplementary Figure 2.** Sequence of pPAM-*GnTI*-KO comprised between left and right borders. LB and RB, *nptII*, SAR, *pcoCas9*, *GnTI*.PTG.

TGGCAGGATATATTGTGGTGTAAACAAATTGACGCTTAGACAACTTAATAACACATTGCGGACGTTTTTAATGTACTGAATTAACGCCGAATTGAATTCATAACTTCGTATAATGTATGCTATACGAAGTTATGGATCCATTTAAATTCTAGAGGCGCGCCCACCGGTGAATTCGCCTCTATCATAGATGTCGCTATAAACCTATTCAGCACAATATATTGTTTTCATTTTAATATTGTACATATAAGTAGTAGGGTACAATCAGTAAATTGAACGGAGAATATTATTCATAAAAATACGATAGTAACGGGTGATATATTCATTAGAATGAACCGAAACCGGCGGTAAGGATCTGAGCTACACATGCTCAGGTTTTTTACAACGTGCACAACAGAATTGAAAGCAAATATCATGCGATCATAGGCGTCTCGCATATCTCATTAAAGCAGCTGGAAGATTTGATGGATCCTCATCAGATCCGAGCTCAAGCTTGGGTCCCGCTCAGAAGAACTCGTCAAGAAGGCGATAGAAGGCGATGCGCTGCGAATCGGGAGCGGCGATACCGTAAAGCACGAGGAAGCGGTCAGCCCATTCGCCGCCAAGCTCTTCAGCAATATCACGGGTAGCCAACGCTATGTCCTGATAGCGGTCCGCCACACCCAGCCGGCCACAGTCGATGAATCCAGAAAAGCGGCCATTTTCCACCATGATATTCGGCAAGCAGGCATCGCCATGGGTCACGACGAGATCCTCGCCGTCGGGCATGCGCGCCTTGAGCCTGGCGAACAGTTCGGCTGGCGCGAGCCCCTGATGCTCTTCGTCCAGATCATCCTGATCGACAAGACCGGCTTCCATCCGAGTACGTGCTCGCTCGATGCGATGTTTCGCTTGGTGGTCGAATGGGCAGGTAGCCGGATCAAGCGTATGCAGCCGCCGCATTGCATCAGCCATGATGGATACTTTCTCGGCAGGAGCAAGGTGAGATGACAGGAGATCCTGCCCCGGCACTTCGCCCAATAGCAGCCAGTCCCTTCCCGCTTCAGTGACAACGTCGAGCACAGCTGCGCAAGGAACGCCCGTCGTGGCCAGCCACGATAGCCGCGCTGCCTCGTCCTGCAGTTCATTCAGGGCACCGGACAGGTCGGTCTTGACAAAAAGAACCGGGCGCCCCTGCGCTGACAGCCGGAACACGGCGGCATCAGAGCAGCCGATTGTCTGTTGTGCCCAGTCATAGCCGAATAGCCTCTCCACCCAAGCGGCCGGAGAACCTGCGTGCAATCCATCTTGTTCAATCCAAGCTCCCATGGATCTGGTTTAAACGTTAACGGATTGAGAGTGAATATGAGACTCTAATTGGATACCGAGGGGAATTTATGGAACGTCAGTGGAGCATTTTTGACAAGAAATATTTGCTAGCTGATAGTGACCTTAGGCGACTTTTGAACGCGCAATAATGGTTTCTGACGTATGTGCTTAGCTCATTAAACTCCAGAAACCCGCGGCTGAGTGGCTCCTTCAACGTTGCGGTTCTGTCAGTTCCAAACGTAAAACGGCTTGTCCCGCGTCATCGGCGGGGGTCATAACGTGACTCCCTTAATTCTCCGCTCATGATCAAGCTTGCGGCCGCGGCGCGCCCGCGGCCGCAAGCTTGCATGCCTGCAAATCGATTTAGTACATAGATGACACCGCGCGCGATAATTTATCCTAGTTTGCGCGCTATATTTTGTTTTCTATCGCGTATTAAATGTATAATTGCGGGACTCTAATCATAAAAACCCATCTCATAAATAACGTCATGCATTACATGTTAATTATTACATGCTTAACGTAATTCAACAGAAATTATATGATAATCATCGCAAGACCGGCAACAGGATTCAATCTTAAGAAACTTTATTGCCAAATGTTTGAACGATCTGCTTGACTCTAGAGGATCAACGTTTGTAGTCGATGGCTTCTGGCTGCTCCAGATATACGGTGGTTTGTGCCGGTTGTGTGCTGGCAATCACCTTGCCGCCACGTACCGAATAACGTACCGGAACCTGACGGCGCAGCGCATCAAACCCATTTTCAGCCGGCAGGATAATCAGGTTGGCGCTGTTTCCGGCGGCAATGCCGTAATCCTGCAAATTCAACGTCCTTGCGCTGTGGTGGGTGATTAAATTCAGGCCATCGTTAATCTGCCCGTAGCCCATCAACTGGCAAACATGCAGCCCCATATGCAGCACTTGCAGCATATTCGCCGTTCCCAGCGGATACCACGGATCGAAGACATCATCGTGACCAAAGCAGACGTTAATGCCGGACTCCAGCATCTCTTTAACGCGCGTGATGCCGCGACGTTTTGGATACGTATCGAAACGTCCTTGCAGATGAATATTGACCAGCGGGTTGGCGACAAAGTTAATACCGGACATTTTCAGCAAGCGGAACAGGCGTGAGGTATACGCCCCGTTATAGGAGTGCATTGCCGTGGTGTGGCTGGCGGTGACTCGCGCGCCCATGCCTTCATGGTGCGCCAGGGCAGCAACGGTTTCGACAAAGCGCGACTGCTCGTCATCGATCTCATCACAGTGAACGTCGATGAGACGGTCGTATTTTTGCGCCAGGGCGAAGGTTTTATGCAGCGACTCCACGCCGTATTCACGGGTAAATTCAAAATGCGGAATCGCCCCCACTACATCTGCCCCTAAGCGTAACGCCTCTTCCAGCAACGCTTCACCGTTGGGATACGACAAAATCCCTTCCTGAGGGAAGGCGACGATTTGCAGATCAATCCACGGCGCGACTTCCTGCTTCACTTCCAGCATTGCTTTCAGCGCAGTTAGCGTTGCATCCGAAACATCGACATGGGTACGCACATGCTGAATGCCGTTGGCAATCTGCCATTTCAGCGTTTGCCATGCGCGTTGTTTCACATCGTCATGGGTTAATAACGCTTTGCGCTCGGCCCAGCGTTCAATGCCTTCAAACAGCGTGCCGGACTGATTCCAGTTCGGTTGTCCGGCGGTTTGCGTGGTGTCCAGGTGAATATGTGGCTCCACAAACGGCGGTATAACTAAACCTTGTTCGGCATCCAGGCTGTTTTCAGTTATGGGCATCACGCCGGATTGCGCATCAATGGCGCTGATTTTTCCGTCCTGCAGATGAATCTGCCACAGCCCCTCTTCGCCTGGTAACCGGGCGTTAATAATTGTTTGTAAAGCGTTATTCGACATCGATAATTGTAAATGTAATTGTAATGTTGTTTGTTGTTTGTTGTTGTTGGTAATTGTTGTAAAAATGAGCTCTTATACTCGAGCGTGTCCTCTCCAAATGAAATGAACTTCATTATATAGAGGAAGGGTCTTGCGAAGGATAGTGGGATTGTGCGTCATCCCTTACGTCAGTGGAGATGTCACATCAATCCACTTGCTTTGAAGACGTGGTTGGAACGTCTTCTTTTTCCACGATGCTCCTCGTGGGTGGGGGTCCATCTTTGGGACCACTGTCGGCAGAGAGATCTTGAATGATAGCCTTTCCTTTATCGCAATGATGGCATTTGTAGGAGCCACCTTCCTTTTTCTACTGTCCTTTCGATGAAGTGACAGATAGCTGGGCAATGGAATCCGAGGAGGTTTCCCGAAATTATCCTTTGTTGAAAAGTCTCAATAGCCCTTTGGTCTTCTGAGACTGTATCTTTGACATTTTTGGAGTAGACCAGAGTGTCGTGCTCCACCATGTTGACGAAGATTTTTCTTCTTGGTCATGAGTCGTAAAAGACTCTGTATGAACTGTTCGCCAGTCTTCACGGCGAGTTCTGTTAGATCCTCGATTTGAATCTTAGACTCCATGCATGGCCTTAGATTCAGTAGGAACTACCTTTTTAGAGACTCCAATCTCTATTACTTGCCTTGGTTTATGAAGCAAGCCTTGAATCGTCCATACTGGAATAGTACTTCTGATCTTGAGAAATATGTCTTTCTCTGTGTTCTTGATGCAATTAGTCCTGAATCTTTTGACTGCATCTTTAACCTTCTTGGGAAGGTATTTGATCTCCTGGAGATTGTTACTCGGGTAGATCGTCTTGATGAGACCTGCTGCGTAGGCCTCTCTAACCATCTGTGGGTCAGCATTCTTTCTGAAATTGAAGAGGCTAACCTTCTCATTATCAGTGGTGAACATAGTGTCGTCACCTTCACCTTCGAACTTCCTTCCTAGATCGTAAAGATAGAGGAAATCGTCCATTGTAATCTCCGGGGCAAAGGAGATCTCTTTTGGGGCTGGATCACTGCTGGGCCTTTTGGTTCCTAGCGTGAGCCAGTGGGCTTTTTGCTTTGGTGGGCTTGTTAGGGCCTTAGCAAAGCTCTTGGGCTTGAGTTGAGCTTCTCCTTTGGGGATGAAGTTCAACCTGTCTGTTTGCTGACTTGTTGTGTACGCGTCAGCTGCTGCTCTTGCCTCTGTAATAGTGGCAAATTTCTTGTGTGCAACTCCGGGAACGCCGTTTGTTGCCGCCTTTGTACAACCCCAGTCATCGTATATACCGGCATGTGGACCGTTATACACAACGTAGTAGTTGATATGAGGGTGTTGAATACCCGATTCTGCTCTGAGAGGAGCAACTGTGCTGTAAGCTCAGATTTTTGTGGGATTGGAATTCACCGGTCTCTCTTAAGGTAGCACCGGTGAATTCCCGATCTAGTAACATAGATGACACCGCGCGCGATAATTTATCCTAGTTTGCGCGCTATATTTTGTTTTCTATCGCGTATTAAATGTATAATTGCGGGACTCTAATCATAAAAACCCATCTCATAAATAACGTCATGCATTACATGTTAATTATTACATGCTTAACGTAATTCAACAGAAATTATATGATAATCATCGCAAGACCGGCAACAGGATTCAATCTTAAGAAACTTTATTGCCAAATGTTTGAACGATCGGGGAAATTCGAGCTCTCACTTGTACAGCTCGTCCATGCCGCCGGTGGAGTGGCGGCCCTCGGCGCGTTCGTACTGTTCCACGATGGTGTAGTCCTCGTTGTGGGAGGTGATGTCCAACTTGATGTTGACGTTGTAGGCGCCGGGCAGCTGCACGGGCTTCTTGGCCTTGTAGGTGGTCTTGACCTCAGCGTCGTAGTGGCCGCCGTCCTTCAGCTTCAGCCTCTGCTTGATCTCGCCCTTCAGGGCGCCGTCCTCGGGGTACATCCGCTCGGAGGAGGCCTCCCAGCCCATGGTCTTCTTCTGCATTACGGGGCCGTCGGAGGGGAAGTTGGTGCCGCGCAGCTTCACCTTGTAGATGAACTCGCCGTCCTGCAGGGAGGAGTCCTGGGTCACGGTCACCACGCCGCCGTCCTCGAAGTTCATCACGCGCTCCCACTTGAAGCCCTCGGGGAAGGACAGCTTCAAGTAGTCGGGGATGTCGGCGGGGTGCTTCACGTAGGCCTTGGAGCCGTACATGAACTGAGGGGACAGGATGTCCCAGGCGAAGGGCAGGGGGCCACCCTTGGTCACCTTCAGCTTGGCGGTCTGGGTGCCCTCGTAGGGGCGGCCCTCGCCCTCGCCCTCGATCTCGAACTCGTGGCCGTTCACGGAGCCCTCCATGTGCACCTTGAAGCGCATGAACTCCTTGATGATGGCCATGTTATCCTCCTCGCCCTTGCTCACCATATTAAGCCTCAGCCTGCAGGTCGTCCTCTCCAAATGAAATGAACTTCCTTATATAGAGGAAGGGTCTTGCGAAGGATAGTGGGATTGTGCGTCATCCCTTACGTCAGTGGAGATATCACATCAATCCACTTGCTTTGAAGACGTGGTTGGAACGTCTTCTTTTTCCACGATGCTCCTCGTGGGTGGGGGTCCATCTTTGGGACCACTGTCGGCAGAGGCATCTTGAACGATAGCCTTTCCTTTATCGCAATGATGGCATTTGTAGGTGCCACCTTCCTTTTCTACTGTCCTTTTGATGAAGTGACAGATAGCTGGGCAATGGAATCCGAGGAGGTTTCCCGATATTACCCTTTGTTGAAAAGTCTCAATAGCCCTTTGGTCTTCTGAGACTGTATCTTTGATATTCTTGGAGTAGACGAGAGTGTCGTGCTCCACCATGTTATCACATCAATCCACTTGCTTTGAAGACGTGGTTGGAACGTCTTCTTTTTCCACGATGCTCCTCGTGGGTGGGGGTCCATCTTTGGGACCACTGTCGGCAGAGGCATCTTGAACGATAGCCTTTCCTTTATCGCAATGATGGCATTTGTAGGTGCCACCTTCCTTTTCTACTGTCCTTTTGATGAAGTGACAGATAGCTGGGCAATGGAATCCGAGGAGGTTTCCCGATATTACCCTTTGTTGAAAAGTCTCAATAGCCCTTTGGTCTTCTGAGACTGTATCTTTGATATTCTTGGAGTAGACGAGAGTGTCGTGCTCCACCATGTTGACGGATCTCTAGAAGCTTGCGGCCGCCTCTCTTAAGGTAGCGAGCTCTTAATTAATAGGGATAACAGGGTAATGCGGCCGCAAGCTTATTCCATGGCTGCAGGTCCCTTACTCCAAGAATATCAAAGATACAGTCTCAGAAGACCAAAGGGCTATTGAGACTTTTCAACAAAGGGTAATATCGGGAAACCTCCTCGGATTCCATTGCCCAGCTATCTGTCACTTCATCAAAAGGACAGTAGAAAAGGAAGGTGGCACCTACAAATGCCATCATTGCGATAAAGGAAAGGCTATCGTTCAAGATGCCTCTGCCGACAGTGGTCCCAAAGATGGACCCCCACCCACAAGGAGCATCGTGGAAAAAGAAGACGTTCCAACCACGTCTTCAAAGCAAGTGGATTGATGTGATATCTCCACTGACGTAAGGGATGACGCACAATCCCACTATCCTTCGCCCCAAGCTTGGGCCCAAGCTTGGGTCGCGCCCCACGGATGGTATAAGAATAAAGGCATTCCGCGTGCAGGATTCACCCGTTCGCCTCTCACCTTTTCGCTGTACTCTCTCGCCACACACACCCCCTCTCCAGCTCCGTTGGAGCTCCGGACAGCAGCAGGCGCGGGGCGGTCACGTAGTAAGCAGCTCTCGGCTCCCTCTCCCCTTGCTCCGTGGATCCATGGATTACAAGGATGATGATGATAAGGATTACAAGGATGATGATGATAAGATGGCTCCAAAGAAGAAGAGAAAGGTTGGAATCCACGGAGTTCCAGCTGCTGATAAGAAGTACTCTATCGGACTTGACATCGGAACCAACTCTGTTGGATGGGCTGTTATCACCGATGAGTACAAGGTTCCATCTAAGAAGTTCAAGGTTCTTGGAAACACCGATAGACACTCTATCAAGAAGAACCTTATCGGTGCTCTTCTTTTCGATTCTGGAGAGACCGCTGAGGCTACCAGATTGAAGAGAACCGCTAGAAGAAGATACACCAGAAGAAAGAACAGAATCTGCTACCTTCAGGAAATCTTCTCTAACGAGATGGCTAAGGTTGATGATTCTTTCTTCCACAGACTTGAGGAGTCTTTCCTTGTTGAGGAGGATAAGAAGCACGAGAGACACCCAATCTTCGGAAACATCGTTGATGAGGTTGCTTACCACGAGAAGTACCCAACCATCTACCACCTTAGAAAGAAGTTGGTTGATTCTACCGATAAGGCTGATCTTAGACTTATCTACCTTGCTCTTGCTCACATGATCAAGTTCAGAGGACACTTCCTTATCGAGGGAGACCTTAACCCAGATAACTCTGATGTTGATAAGTTGTTCATCCAGCTTGTTCAGACCTACAACCAGCTTTTCGAGGAGAACCCAATCAACGCTTCTGGAGTTGATGCTAAGGCTATCCTTTCTGCTAGACTTTCTAAGTCTCGTAGACTTGAGAACCTTATCGCTCAGCTTCCAGGAGAGAAGAAGAACGGACTTTTCGGAAACCTTATCGCTCTTTCTCTTGGACTTACCCCAAACTTCAAGTCTAACTTCGATCTTGCTGAGGATGCTAAGTTGCAGCTTTCTAAGGATACCTACGATGATGATCTTGATAACCTTCTTGCTCAGATCGGAGATCAGTACGCTGATCTTTTCCTTGCTGCTAAGAACCTTTCTGATGCTATCCTTCTTTCTGACATCCTTAGAGTTAACACCGAGATCACCAAGGCTCCACTTTCTGCTTCTATGATCAAGAGATACGATGAGCACCACCAGGATCTTACCCTTTTGAAGGCTCTTGTTAGACAGCAGCTTCCAGAGAAGTACAAGGAAATCTTCTTCGATCAGTCTAAGAACGGATACGCTGGATACATCGATGGAGGAGCTTCTCAGGAGGAGTTCTACAAGTTCATCAAGCCAATCCTTGAGAAGATGGATGGAACCGAGGAGCTTCTTGTTAAGTTGAACAGAGAGGATCTTCTTAGAAAGCAGAGAACCTTCGATAACGGATCTATCCCACACCAGATCCACCTTGGAGAGCTTCACGCTATCCTTCGTAGACAGGAGGATTTCTACCCATTCTTGAAGGATAACAGAGAGAAGATCGAGAAGATCCTTACCTTCAGAATCCCATACTACGTTGGACCACTTGCTAGAGGAAACTCTCGTTTCGCTTGGATGACCAGAAAGTCTGAGGAGACCATCACCCCTTGGAACTTCGAGGAGGTAAGTTTCTGCTTCTACCTTTGATATATATATAATAATTATCATTAATTAGTAGTAATATAATATTTCAAATATTTTTTTCAAAATAAAAGAATGTAGTATATAGCAATTGCTTTTCTGTAGTTTATAAGTGTGTATATTTTAATTTATAACTTTTCTAATATATGACCAAAATTTGTTGATGTGCAGGTTGTTGATAAGGGAGCTTCTGCTCAGTCTTTCATCGAGAGAATGACCAACTTCGATAAGAACCTTCCAAACGAGAAGGTTCTTCCAAAGCACTCTCTTCTTTACGAGTACTTCACCGTTTACAACGAGCTTACCAAGGTTAAGTACGTTACCGAGGGAATGAGAAAGCCAGCTTTCCTTTCTGGAGAGCAGAAGAAGGCTATCGTTGATCTTCTTTTCAAGACCAACAGAAAGGTTACCGTTAAGCAGTTGAAGGAGGATTACTTCAAGAAGATCGAGTGCTTCGATTCTGTTGAAATCTCTGGAGTTGAGGATAGATTCAACGCTTCTCTTGGAACCTACCACGATCTTTTGAAGATCATCAAGGATAAGGATTTCCTTGATAACGAGGAGAACGAGGACATCCTTGAGGACATCGTTCTTACCCTTACCCTTTTCGAGGATAGAGAGATGATCGAGGAGAGACTCAAGACCTACGCTCACCTTTTCGATGATAAGGTTATGAAGCAGTTGAAGAGAAGAAGATACACCGGATGGGGTAGACTTTCTCGTAAGTTGATCAACGGAATCAGAGATAAGCAGTCTGGAAAGACCATCCTTGATTTCTTGAAGTCTGATGGATTCGCTAACAGAAACTTCATGCAGCTTATCCACGATGATTCTCTTACCTTCAAGGAGGACATCCAGAAGGCTCAGGTTTCTGGACAGGGAGATTCTCTTCACGAGCACATCGCTAACCTTGCTGGATCTCCAGCTATCAAGAAGGGAATCCTTCAGACCGTTAAGGTTGTTGATGAGCTTGTTAAGGTTATGGGTAGACACAAGCCAGAGAACATCGTTATCGAGATGGCTAGAGAGAACCAGACCACCCAGAAGGGACAGAAGAACTCTCGTGAGAGAATGAAGAGAATCGAGGAGGGAATCAAGGAGCTTGGATCTCAAATCTTGAAGGAGCACCCAGTTGAGAACACCCAGCTTCAGAACGAGAAGTTGTACCTTTACTACCTTCAGAACGGAAGAGATATGTACGTTGATCAGGAGCTTGACATCAACAGACTTTCTGATTACGATGTTGATCACATCGTTCCACAGTCTTTCTTGAAGGATGATTCTATCGATAACAAGGTTCTTACCCGTTCTGATAAGAACAGAGGAAAGTCTGATAACGTTCCATCTGAGGAGGTTGTTAAGAAGATGAAGAACTACTGGAGACAGCTTCTTAACGCTAAGTTGATCACCCAGAGAAAGTTCGATAACCTTACCAAGGCTGAGAGAGGAGGACTTTCTGAGCTTGATAAGGCTGGATTCATCAAGAGACAGCTTGTTGAGACCAGACAGATCACCAAGCACGTTGCTCAGATCCTTGATTCTCGTATGAACACCAAGTACGATGAGAACGATAAGTTGATCAGAGAGGTTAAGGTTATCACCTTGAAGTCTAAGTTGGTTTCTGATTTCAGAAAGGATTTCCAGTTCTACAAGGTTAGAGAGATCAACAACTACCACCACGCTCACGATGCTTACCTTAACGCTGTTGTTGGAACCGCTCTTATCAAGAAGTACCCAAAGTTGGAGTCTGAGTTCGTTTACGGAGATTACAAGGTTTACGATGTTAGAAAGATGATCGCTAAGTCTGAGCAGGAGATCGGAAAGGCTACCGCTAAGTACTTCTTCTACTCTAACATCATGAACTTCTTCAAGACCGAGATCACCCTTGCTAACGGAGAGATCAGAAAGAGACCACTTATCGAGACCAACGGAGAGACCGGAGAGATCGTTTGGGATAAGGGAAGAGATTTCGCTACCGTTAGAAAGGTTCTTTCTATGCCACAGGTTAACATCGTTAAGAAAACCGAGGTTCAGACCGGAGGATTCTCTAAGGAGTCTATCCTTCCAAAGAGAAACTCTGATAAGTTGATCGCTAGAAAGAAGGATTGGGACCCAAAGAAGTACGGAGGATTCGATTCTCCAACCGTTGCTTACTCTGTTCTTGTTGTTGCTAAGGTTGAGAAGGGAAAGTCTAAGAAGTTGAAGTCTGTTAAGGAGCTTCTTGGAATCACCATCATGGAGCGTTCTTCTTTCGAGAAGAACCCAATCGATTTCCTTGAGGCTAAGGGATACAAGGAGGTTAAGAAGGATCTTATCATCAAGTTGCCAAAGTACTCTCTTTTCGAGCTTGAGAACGGAAGAAAGAGAATGCTTGCTTCTGCTGGAGAGCTTCAGAAGGGAAACGAGCTTGCTCTTCCATCTAAGTACGTTAACTTCCTTTACCTTGCTTCTCACTACGAGAAGTTGAAGGGATCTCCAGAGGATAACGAGCAGAAGCAGCTTTTCGTTGAGCAGCACAAGCACTACCTTGATGAGATCATCGAGCAAATCTCTGAGTTCTCTAAGAGAGTTATCCTTGCTGATGCTAACCTTGATAAGGTTCTTTCTGCTTACAACAAGCACAGAGATAAGCCAATCAGAGAGCAGGCTGAGAACATCATCCACCTTTTCACCCTTACCAACCTTGGTGCTCCAGCTGCTTTCAAGTACTTCGATACCACCATCGATAGAAAAAGATACACCTCTACCAAGGAGGTTCTTGATGCTACCCTTATCCACCAGTCTATCACCGGACTTTACGAGACCAGAATCGATCTTTCTCAGCTTGGAGGAGATAAGAGACCAGCTGCTACCAAGAAGGCTGGACAGGCTAAGAAGAAGAAGTGACTGCAGATCGTTCAAACATTTGGCAATAAAGTTTCTTAAGATTGAATCCTGTTGCCGGTCTTGCGATGATTATCATATAATTTCTGTTGAATTACGTTAAGCATGTAATAATTAACATGTAATGCATGACGTTATTTATGAGATGGGTTTTTATGATTAGAGTCCCGCAATTATACATTTAATACGCGATAGAAAACAAAATATAGCGCGCAAACTAGGATAAATTATCGCGCGCGGTGTCATCTATGTTACTAGATCAGGGACGAATTCACCGGTTAGGGATAACAGGGTAATTAACTATAACGGTCCTAAGGTAGCGAGCGGCCGCAAGCTTATTCCATGGCTGCAGGAGAAATCTCAAAATTCCGGCAGAACAATTTTGAATCTCGATCCGTAGAAACGAGACGGTCATTGTTTTAGTTCCACCACGATTATATTTGAAATTTACGTGAGTGTGAGTGAGACTTGCATAAGAAAATAAAATCTTTAGTTGGGAAAAAATTCAATAATATAAATGGGCTTGAGAAGGAAGCGAGGGATAGGCCTTTTTCTAAAATAGGCCCATTTAAGCTATTAACAATCTTCAAAAGTACCACAGCGCTTAGGTAAAGAAAGCAGCTGAGTTTATATATGGTTAGAGACGAAGTAGTGATTGAACAAAGCACCAGTGGTCTAGTGGTAGAATAGTACCCTGCCACGGTACAGACCCGGGTTCGATTCCCGGCTGGTGCACGACAGCAGCCAAGATGAGGGTTTCAGAGCTATGCTGGAAACAGCATAGCAAGTTGAAATAAGGCTAGTCCGTTATCAACTTGAAAAAGTGGCACCGAGTCGGTGCAACAAAGCACCAGTGGTCTAGTGGTAGAATAGTACCCTGCCACGGTACAGACCCGGGTTCGATTCCCGGCTGGTGCAAATCTGGTCAATAAGCAATCGTTTCAGAGCTATGCTGGAAACAGCATAGCAAGTTGAAATAAGGCTAGTCCGTTATCAACTTGAAAAAGTGGCACCGAGTCGGTGCAACAAAGCACCAGTGGTCTAGTGGTAGAATAGTACCCTGCCACGGTACAGACCCGGGTTCGATTCCCGGCTGGTGCAGAAAGCATCCATCCAAGACCGTTTCAGAGCTATGCTGGAAACAGCATAGCAAGTTGAAATAAGGCTAGTCCGTTATCAACTTGAAAAAGTGGCACCGAGTCGGTGCAACAAAGCACCAGTGGTCTAGTGGTAGAATAGTACCCTGCCACGGTACAGACCCGGGTTCGATTCCCGGCTGGTGCAAGACTCAAAGAGAATCACAGGTTTCAGAGCTATGCTGGAAACAGCATAGCAAGTTGAAATAAGGCTAGTCCGTTATCAACTTGAAAAAGTGGCACCGAGTCGGTGCTTTTTTTCTAGACCCAGCTTTCTTGTACAAAGTTGGCATTAATCACTAGTGAATTCACCGGTTAACTATAACGGTCCTAAGGTAGCGATGGCAAACAGCTATTATGGGTAGCGGCCGCAAGCTTAGAAATCTCAAAATTCCGGCAGAACAATTTTGAATCTCGATCCGTAGAAACGAGACGGTCATTGTTTTAGTTCCACCACGATTATATTTGAAATTTACGTGAGTGTGAGTGAGACTTGCATAAGAAAATAAAATCTTTAGTTGGGAAAAAATTCAATAATATAAATGGGCTTGAGAAGGAAGCGAGGGATAGGCCTTTTTCTAAAATAGGCCCATTTAAGCTATTAACAATCTTCAAAAGTACCACAGCGCTTAGGTAAAGAAAGCAGCTGAGTTTATATATGGTTAGAGACGAAGTAGTGATTGAACAAAGCACCAGTGGTCTAGTGGTAGAATAGTACCCTGCCACGGTACAGACCCGGGTTCGATTCCCGGCTGGTGCATCTCTTCAGATGTTCCTGTGTTTCAGAGCTATGCTGGAAACAGCATAGCAAGTTGAAATAAGGCTAGTCCGTTATCAACTTGAAAAAGTGGCACCGAGTCGGTGCAACAAAGCACCAGTGGTCTAGTGGTAGAATAGTACCCTGCCACGGTACAGACCCGGGTTCGATTCCCGGCTGGTGCATCTCTTCGGATGTTCCTGTGTTTCAGAGCTATGCTGGAAACAGCATAGCAAGTTGAAATAAGGCTAGTCCGTTATCAACTTGAAAAAGTGGCACCGAGTCGGTGCAACAAAGCACCAGTGGTCTAGTGGTAGAATAGTACCCTGCCACGGTACAGACCCGGGTTCGATTCCCGGCTGGTGCATTCTCTGTTTTAGGTTGTACGTTTCAGAGCTATGCTGGAAACAGCATAGCAAGTTGAAATAAGGCTAGTCCGTTATCAACTTGAAAAAGTGGCACCGAGTCGGTGCAACAAAGCACCAGTGGTCTAGTGGTAGAATAGTACCCTGCCACGGTACAGACCCGGGTTCGATTCCCGGCTGGTGCATTCTCAGTTTTAGGTTGCACGTTTCAGAGCTATGCTGGAAACAGCATAGCAAGTTGAAATAAGGCTAGTCCGTTATCAACTTGAAAAAGTGGCACCGAGTCGGTGCAACAAAGCACCAGTGGTCTAGTGGTAGAATAGTACCCTGCCACGGTACAGACCCGGGTTCGATTCCCGGCTGGTGCAGCCGCTTTCATTTCTAATTGGTTTCAGAGCTATGCTGGAAACAGCATAGCAAGTTGAAATAAGGCTAGTCCGTTATCAACTTGAAAAAGTGGCACCGAGTCGGTGCAACAAAGCACCAGTGGTCTAGTGGTAGAATAGTACCCTGCCACGGTACAGACCCGGGTTCGATTCCCGGCTGGTGCAGCTGCTTTTATTTCTAATTGGTTTCAGAGCTATGCTGGAAACAGCATAGCAAGTTGAAATAAGGCTAGTCCGTTATCAACTTGAAAAAGTGGCACCGAGTCGGTGCTTTTTTTCTAGACCCAGCTTTCTTGTACAAAGTTGGCATTAGAATATATATATATTCCCAAGCTGAATTCACCGGTTGGCAAACAGCTATTATGGGTATTATGGGTTTATGGGTGGTTCTTTATGCGGACACTGACGGCTTTATGCCTGCAGGTCGCGAGCGATCGCGGTACCGCCCGGGCGTCGACATAACTTCGTATAATGTATGCTATACGAAGTTATAAGCTTAGCTTGAGCTTGGATCAGATTGTCGTTTCCCGCCTTCAGTTTAAACTATCAGTGTTTGACAGGATATATTGGCGGGTAAAC

**Supplementary Figure 3.** Sequence of pPZP-*GnTI*/*FucT*-KO comprised between left and right borders. LB and RB, *nptII*, *codA1*, mCherry, *pcoCas9*, *GnTI*.PTG, *FucT*.PTG, homing endonuclease site.

ATGATATGGAAATTGCCCCTGATTTTTTTGACTTTTTTGAGGCTGGAGCTACTCTTCTTGACAGAGACAAGTAAGGCCGGATGTTGCGTTGTTTTACTTTCAAAGTGTGAGAATGCACGGGAGAAAATATTATTGATATTATTCTCTTATATTAAACACAATACAATGAGCCCTATATATATATATATATATATATATACATAACATGTCCTACTCCTAATACATATGAGATTAGGGTTATTTACTCCTATTTACATAACTATTCTAATACTTCCCCTCAAGATGGTGCATATAAATCATATGTACCGAGCTTGTTACATATGTAGTTAATACGAGGACCAGTGAGGGACTTGGTGAAAATATCTGCAAGCTGATCATTCGACTTCACAAACTTTGTAGCAATATCTCCCGAGAATATCTTTTCTCTCACAAAGTGACAGTCAATCTCAATGTGTTTAGTTCTCTCATGAACACTGGATTTGACGCAATATGAAGAGCAACTTGATTATCACACACAAGTCCCATCTGACTAATCACACCAAATTTCAACTCCTTGAGAAACTGTTTGATCCAAATTAGCTCACATGTCGCCATAGCCATCGCTCGATATTCTGCTTCTGCACTAGACCGAGCAACCACATTTTGTTTCTTGCTCTTCCAGAACAACAAATTTCCTCCTACTAAGATACAATATCCAGACGTAGAACGCCTATCAGAAGGTGATCCTGCCCAATCAGCATCTGAGTATCCAACGATCTGCTCATGGCCTCGATCCTCAAACAATAAACCTTTGCCTGGAGCTGATTTTATATACCGAAGAATGCAAACAACTGCATCCCAATGACTATCACACGGAGAATCCATAAACTGACTCACAACATTCACAGAAAAGGAAATGTCAGGTCTTGTCACTGTAAGGTAATTTAATTTACCAACCAACCGTCTATATCTTGCAGGATCGCTAAGAGACTCCCCCTGTTCTGGCAGAAGTTTAGAATTCGGATCCATCGGAGTGTCAACAGGTCTACAACCTGTCATTCCTGTCTCCTCAAGAATATCTAAGGCATACTTTCGTTGTGAGATCACAATACCTGAGCTAGACTGAGCGACCTCAATACCCAGAAAATACTTTAGTTTGCCCAGATCCTTAGTCTGAAAGTGCTGAAAGAGATGTTGCTTCAATTTAGTAATACTATCCTGGTCATTGCCGGTAATAACAATGTCGTCAACATAGACCACCAGATAAATACAGAGATCCGAAGCAGAATGCCGATAGAATACTGAGTGATCAGTTTCACTCCGAATCATGCCAAATTCCTGGATAACTGTGCTGAACTTACCAAACCAGACTCGAAGAGACTGCTTTAGACCATAAAGTGACCGGCGCAAGCGACATACAAGGCCACGAGACTCCCCCTGAGCAACAAACCCAGGTGGTTGCTTCATATAAACTTCATTCACAAGGTCACCGTGTAAAAATGCATTCTTAATGTCCCGCTGATAGAGGGGCCAATGGCGAACAACAACCATGGATAGAAAAAGGCGAACTGATGCTATCTTAGCCACGGGGGAGAAGGTATCACTGTAATCGAGCCCAAATATTTGAGTATATCCCTTGGCAACAAGACGAGCCTTAAGACGATCAACCTTACCATCCAGACCAACCTTGACTGCATACACCCAACGACAACCAACAGAAGATTTACCTGAAGAAAGAGGGACGAGCTCCCAAGTACCACTCGTATGTAAAGCAGACATCTCGTCAATCATAGCCTGTCGCCATCCTAGATGAGACAATGCTTCACCTATAGACTTAGGGATGGAAACAGAGGACAAAAAAGATAGAAAAGCATAATAGGGTGATGACAAACGATGATAACTCAAACCGACATAATGGGGATTAGGATTAAGGGTGGTCCGTATACCTTTCCGAAGTGCAATCGGTGTACTAGGAAGAGACAAGTCCGCAGGAGGAGCATGGTCAGGTGCAGGACGTGAAATCAGCTGGGCCTGATGCTGGGTGCGGACGACGATGATATGTCAAGAGTGGTGTTCCTGTGGCAGGGAATCTAGGAGGAGCCACACTAGACTCCCCAACGGTTGGAATGGGTAAGACTTTTGTAGCGGAAGGTGAAGGAGGAGCTATAGTAGACTCGGGTAAGACTTCTGTGGCGGAAAGTGAAGGAGGAGCTATAGTAGACTCCTTAAAGGTCGGTATAGGTAAGACCTCAGATATATCAAGGTGGTCAGAATGGTTAGAAAAGGTAAAGAAAGGTTTAGACTCAAAAAATGTGACGTCAGAGGACATAAAGTACCTACGAATATCAGGTGAGTAACAACGATATCCCTTCTGAACACGAGAATAACCAAGGAAGACACACTTGAGAGCACGATGAGCTAACTTATCTTTCCCAGGGGCTAAGTTATGAACGAAACAAGTGCTCCCAAAAACACGAGGAGGAACAGAGTATAAAGGTGATTGGGGAAACAATACTGCATACGGTATCTGATTGTGAATGGGAGAGGAAGGCATCCGATTAACTAAATAACAAGCTGTGAGAACTGCATCGCCCCAAAAACGCAACGGAATATGAGATTCAATGAGAAGTGTGCGAGCAGTCTCAATGATGTGCCTATTCTTTCTCTCTGCAACCCCATTTTGCTAAAGGGTATAAGGACAAGAGGTCTAATGAATAATTCCTTAAGAAGTCATAAACTGCTGAAATTGAGAGGATAAATATTCTAAGGCATTATCACTGCGAAAAGTGCGAATAGAAACACCAAATTGATTTTTAATTTCAGCACAAAAATTCTGGAATATAGAAAACAACTCAGAACGATCTTTCATTAAGAAAATCCAAGTACATCTTGCATGATCATCAATGAAACTAACAAAATAACGAAATCCCAAGGTTGAACTGACTCTACTAGGACCCCATATATCAGAATGAACTAAAGAAAAAACAGACTATGCATGACTCTCAATACTACGAGGAAAGGAGGCTCGGGTATGTTTCCCGAGCTGACATGACTCACACTCTAGTGTAGATAAACTAGACAAACTAGGCACCATCTTCTGAAGCTTGGATAAGCTTGGATGTCCTAAACGTCTATGAATTAGGTCCGGAGTATCTGTAACTAGACATGCCTTGGAGGAATTGAGTGAGTTAAGGTAGTAAAGGCCTTCTGATTCAAGTCCTGTTCCAATCGTCTGCCCCGTACTGCGGTCCTGCATAATAAAGGAATCATCAATAAAATATATACCACAATGGAGGGCACGAGTCAAACGACTAACAAATGCAAGATTAAAAGGACATCCAGGGACATAGAGAACGGAATCTAGAGTGACAGAGGGTAGGGGATTCGCTTGTCCAACTCCTTTTGCTTTGGTTTGAGACCCATTGGCTAAAGTAACAGTGGGAAGAGACTGTGAATACGCAATATTTGACAAATGTGATTTATTACCAGAGATATGATCAGAAGCGGCCGAGTCCACAACCCATTGTCCAAGAGTACTAGACTGGAAAACACAAACAAAAGAATTACCAGCAATAGAAGTATCAGTCTGAGCAACAGAGGCTATTTGTGGAGATGTCTGCTTACTTGCTCGATACTGAAGGAACTCATTATACTCCCCTTCAGATAAAGAAAAACCCTGGTTACCTGTAGTCTCAGTCTGAGCATGTAAAAAATAGCACACGTCACAAGTGTGTCCAAGTTTATGACAATAAGAGCACTTGGGTCTAGATCTTCCAAAACGACATCCTCCTCGTCTATTCTCCATAGTATGAGATGCCCGATTGTCCACTGACTGGGATACGAGGACAGATGAGTCAAGTGTCTGTGATGAGCTCACTGGGTGACTTGGTGCTGCAGCAAGGCGAAGTAATCGAGAGAATAATTCATCAACTGTGGGGACAGTCGGACTAGCCAAAATCTGGTCACGTACTGAATCAAGGTCATTAGGGAGTCCAGCGAGTGTAAGAACTAGAAACATCTTCTGTCGTTGCTCTTGTTGCTTTTCAATACTAGCAGAAACTGGTATCAATGTCTCAAATTCTTCCATGACTGCATGTACTTGTCCCAAGTAAGTAGACATATCCAATTCCTGTTTCTTCAAGCTTGTCATTCGCGATATTACATCATAGAAACGAGATATGTCATTAGTGTATAAATTACGAGCCTTTTCCCAAACTAAATAACATCTCTGGAATGGACGGAACAAGGGCATCAACTTGGAATCAATAGATCGCCACAGGATACTACATAACTGAGCATCGACCTTCTCCCACAGTGTTTTGGCCTTTTCATCACCTTCGCTAGCCTTTTTTATTAAATGATCTTGAACTCCTTGACCTTTACACCACAACTCGACAGACGAAGCCCAAGCTAAGTAGTGTGAACCTCCCATTAAAGGTTCTGAGGTAATCATAACACCGGAACTGCCAGAACCCGGTTTTTTAGATCCGAAAGCATCGACTCCCAAATACATCGTGAAAACTCGCTGAAACAGATGAAGGAAACACTGTTTTTGCCGGGAAATTACTGTAGCTGCCGGAAAAACCTCAAAATGGTCGGAATCAAACGAAAAGAGGATGGGTAGGGTCAGAATTAGTAGCCGACCCAACTGTTCTGAAGATCCGTCGTCAAAAAATGGCCGGAAAGGGCTCCACTCGCCGGCGCGTGAAGCAGATCTCGCCGGAAAAAAAATCCTCCGGGCGGCGCGTGGCGGCGCGTGAGAGGTTGTCTGCCGGAGGTGTTTGATGGGGTTTGGTCGCCGGGGGTTGGGGGATGTTGTGGTGGTGTTGGTTTTTGCACAACACCGATGGAAATTGATTTTTTCTCGAAAAACAGCCCTAAAAGGTCGTCGGAATTGATGCACGATGACTGTTTTGGGTGGGTTTTCTTTCCCGGATATTTTCTCACTGCTGCTCTGATACCATGTGAGAATGCACGGGAGAAAATATTATTGATATTATTCTCTTATATTAAACACAATACAATGAGCCCTATATATATATATATATATACATAACATGTCCTACTCCGAATACATATGGGATTAGGGTTATTTACTCCTATTTACATAACTATTCTAACACAAAGAATTATTCAATTTATCCTAGTCTGAGGAAAATTACTATTTTTTTACTCGTGTCCAACTCCCCCCTCATTTTCTTTAAAGAACCAACATAATCAGATTCGACAGCATCCAAGATCTCCTGCTCTTCCAGGCTTGTGATAGGAGAAAATCTGATTGCAGCGAGGGGGATAGATTGATTTCCATTTTGGTTATATAACATTCTTAGCAAAAGGATTAAAAGCTTTTCCCTCATGGACTGATGTCCAAATATGCTAAGTGGTATAGTGAACGAACTAGAATGGGATTAGCCTAAAACATGGGGATAAAAAGCCTGTTCTAAATGTCCCAAGTATGTTACAAGAATTTCCTAAATTGTTATGGTGAACATCCCAGGTCGATTATGGCTATTTCTTCTTGGAATGACAATGGACAAATGCAGTTTGTCCAAGATCCTT

**Supplementary Figure 4.** Sequence of *NtGnTI.B* intron 11. Exon 11, Exon 12, transposon-related ORFs.


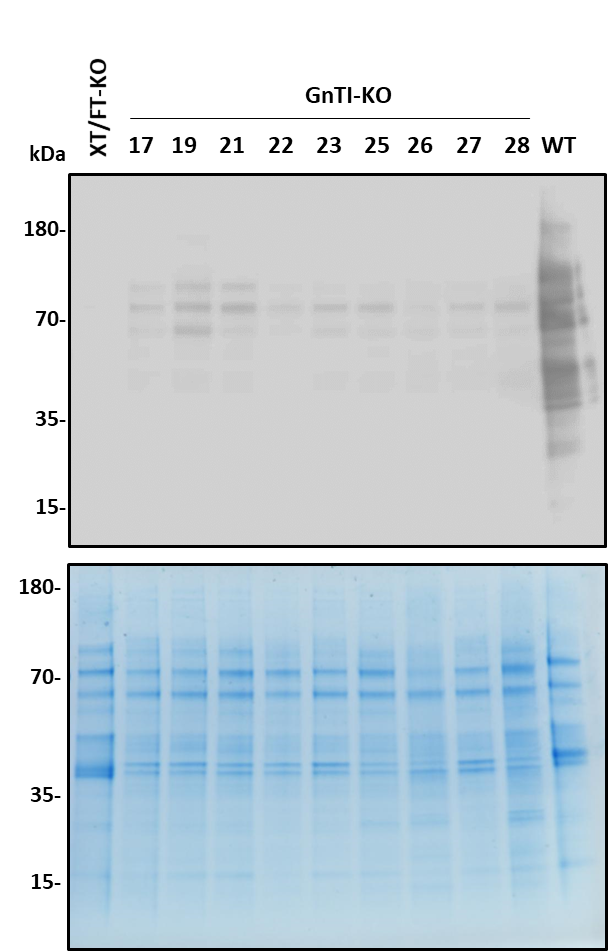


**Supplementary Figure 5**. Reduction of protein-associated β1,2-xylose and α1,3-fucose residues in *GnTI*-KO BY-2 cell lines. Top. One representative Western blotting membrane is shown. Secreted proteins (40 µL culture medium) from WT, *XylT*/*FucT*-KO, and from the indicated *GnTI*-KO cell lines were separated by gel electrophoresis and analyzed by Western blotting using anti-β1,2-xylose and anti-α1,3-fucose antibodies. Bottom. A colloidal blue gel is displayed as a loading control.

**
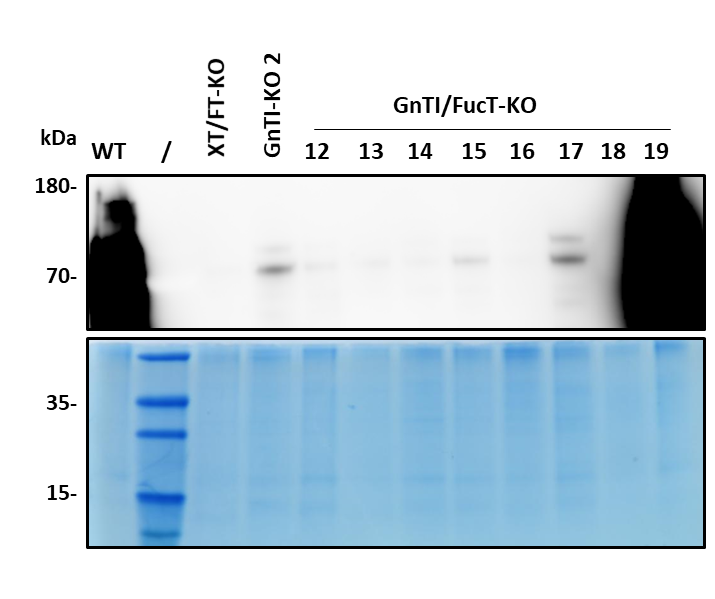
**

**Supplementary Figure 6.** Extensive reduction of complex N-glycan-associated signal on secreted glycoproteins from the indicated GnTI/FucT-KO BY-2 cell lines. One representative Western blotting membrane is shown. Secreted proteins (40 µL culture medium) from WT, *XylT*/*FucT*-KO, *GnTI*-KO#2, and from the indicated *GnTI*/*FucT*-KO cell lines were separated by gel electrophoresis. The gel was horizontally cut and the upper half was analyzed by Western blotting using anti-β1,2-xylose and anti-α1,3-fucose antibodies. The lower half was stained with colloidal blue and is displayed as a loading control. Lane 2 contains a protein ladder.


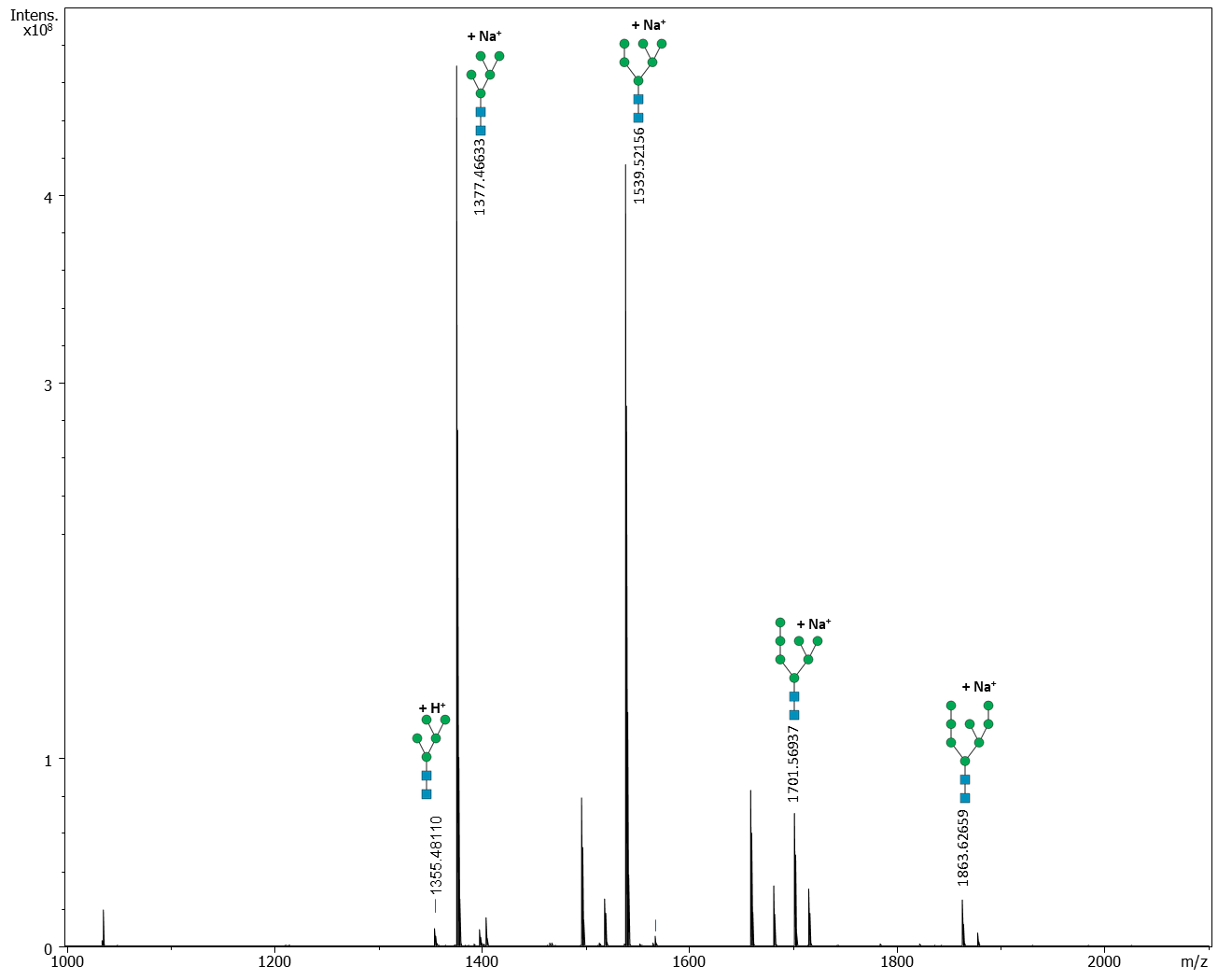


**Supplementary Figure 7**. Total N-glycans released from RNAse B. Peaks identified as N-glycan signals were labeled. Signals were detected as singly positively charged [M+H]^+^ or [M+Na]^+^.

## Supplementary Tables

**Supplementary Table 1.** Primers used in this study. The name of a primer indicates if it is specific to *GnTI.A* (A) or *GnTI.B* (B) or if it is not ortholog-specific (no A/B prefix), which intron (In#) or exon (Ex#) it hybridizes to, and its sense: forward (F) or reverse (R).

| **Name** | **Sequence (5‘-3‘)** |
| --- | --- |
| AEx1F | CCTTCAATCGAAATCGCACG |
| BEx1F | CCTTCAATCGAATTCGCACG |
| Ex6F | ACCTGGAAAAGACTATTAAATCCATC |
| BIn8F | CCTCTCTCTCCATAAGTCC |
| BIn12R | CAAATATTCAATCGAAGCCCC |
| Ex15R | TTAATTGGCTCAAGATACTGCTTG |
| AEx19R | CACTTGGCTTAAAAAAAATAGGTTGTGA |
| BEx19R | TGTTCTGAACACTTAAAATAATAGGTTGTGT |
| Ex1F | AGACTATTCGCTTTCTCCTAAA |
| In3R | GGTATTGGTTAGCTCATGC |
| In12F | GTATATCCTCCATGGAGGT |

**Supplementary Table 2.** Characteristics of crRNAs used to inactivate *NtGnTI* genes.

| **Name** | **Sequence (5‘-3‘)** | **Sense** | **Exon targeted** |
| --- | --- | --- | --- |
| gRNA1 | CGACAGCAGCCAAGATGAGG | Reverse | 1 |
| gRNA2 | AATCTGGTCAATAAGCAATC | Reverse | 3 |
| gRNA3 | GAAAGCATCCATCCAAGACC | Reverse | 13 |
| gRNA4 | AGACTCAAAGAGAATCACAG | Forward | 14 |

**Supplementary Table 3.** Relative amounts of total N-glycans (%) of RNAse B. Relative amounts were determined by considering only the absolute intensities of the peaks associated to a N-glycan structure.

| **Composition** | **Putative structure** | **Abbrev.*** | **Rel. Amount** |
| --- | --- | --- | --- |
| HexNAc2Hex8 | 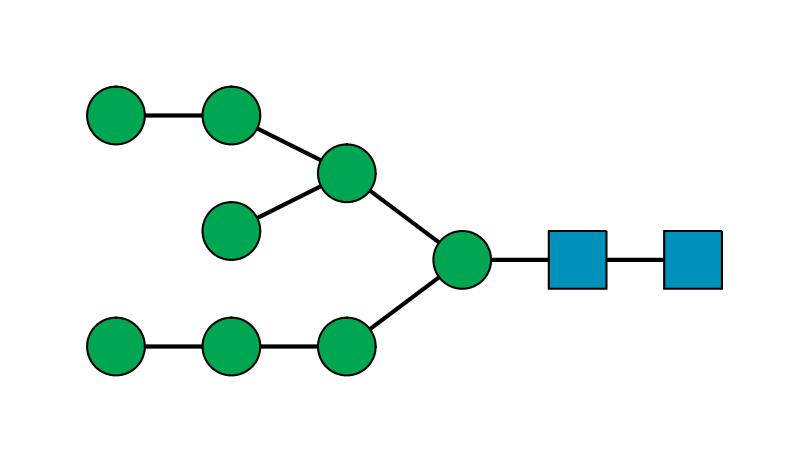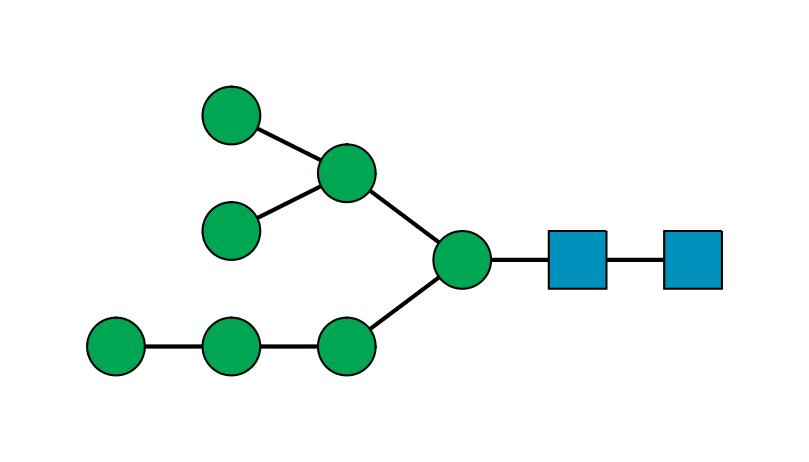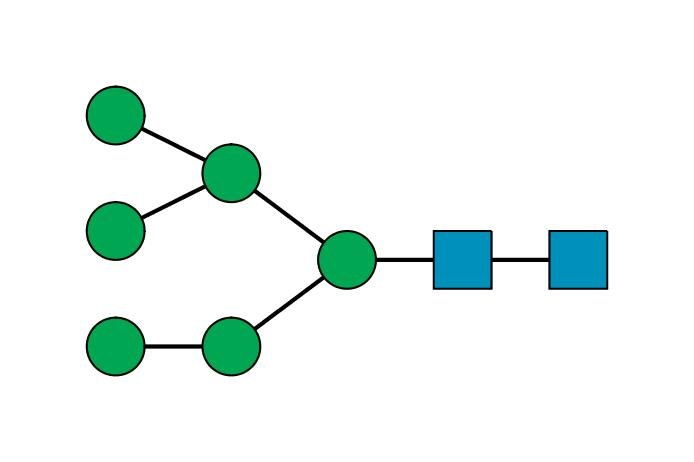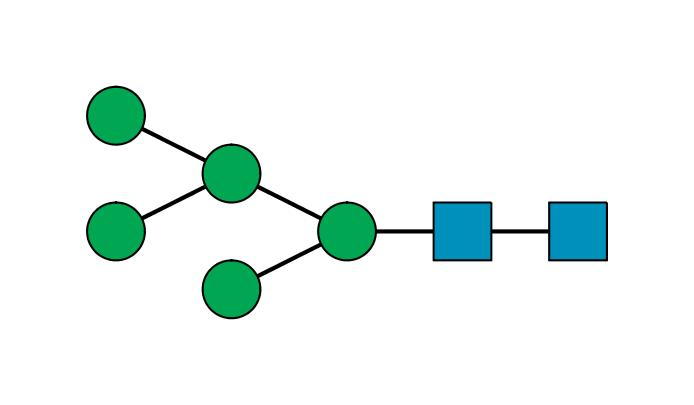 | Man8 | 2.5% |
| HexNAc2Hex7 |  | Man7 | 7.2% |
| HexNAc2Hex6 |  | Man6 | 42.2% |
| HexNAc2Hex5 |  | Man5 | 48.1% |

*According to the nomenclature (Proglycan short) from http://www.proglycan.com.
